# Updated risk-oriented strategy for acute lymphoblastic leukemia in adult patients 18–65 years: NILG ALL 10/07

**DOI:** 10.1038/s41408-020-00383-2

**Published:** 2020-11-13

**Authors:** Renato Bassan, Chiara Pavoni, Tamara Intermesoli, Orietta Spinelli, Manuela Tosi, Ernesta Audisio, Filippo Marmont, Chiara Cattaneo, Erika Borlenghi, Sergio Cortelazzo, Irene Cavattoni, Monica Fumagalli, Daniele Mattei, Claudio Romani, Agostino Cortelezzi, Nicola Fracchiolla, Fabio Ciceri, Massimo Bernardi, Anna Maria Scattolin, Lorella Depaoli, Arianna Masciulli, Elena Oldani, Alessandro Rambaldi

**Affiliations:** 1 U.O.C. Ematologia, Ospedale dell’Angelo, Mestre-Venezia, Italy; 2 U.O.C. Ematologia, Azienda Socio Sanitaria Territoriale Papa Giovanni XXIII, Bergamo, Italy; 3 Ematologia 2, Presidio Ospedaliero Molinette, A.O.U. Città della Salute e della Scienza, Torino, Italy; 4 Divisione di Ematologia, Spedali Civili, Brescia, Italy; 5 Divisione di Ematologia e TMO, Ospedale S. Maurizio, Bolzano, Italy; 6 U.O. di Ematologia e TMO, Ospedale S. Gerardo, Monza Brianza, Italy; 7 S.C. Ematologia, Azienda Ospedaliera S. Croce e Carle, Cuneo, Italy; 8 U.O. Ematologia e Centro TMO, Ospedale Armando Businco, Cagliari, Italy; 9 U.O. Ematologia e TMO, Fondazione IRCSS Cà Granda, Ospedale Maggiore Policlinico, Milano, Italy; 10 Ematologia e TMO, Ospedale S. Raffaele, Milano, Italy; 11 Dipartimento di Ematologia e Medicina Trasfusionale, Azienda Ospedaliera SS. Antonio e Biagio e Cesare Arrigo, Alessandria, Italy; 12 FROM, Fondazione per la Ricerca, Ospedale Papa Giovanni XXIII, Bergamo, Italy; 13 Dipartimento di Oncologia-Ematologia, Università di Milano, Milano, Italy

**Keywords:** Haematological cancer, Haematological diseases, Acute lymphocytic leukaemia

## Abstract

An updated strategy combining pediatric-based chemotherapy with risk-oriented allogeneic hematopoietic cell transplantation (HCT) was evaluated in Philadelphia chromosome-negative acute lymphoblastic leukemia (Ph− ALL) and compared with a published control series. Following induction–consolidation chemotherapy, responsive patients were assigned to receive maintenance chemotherapy or undergo early HCT according to the risk stratification criteria and minimal residual disease (MRD) status. Of the 203 study patients (median age 41 years, range 17–67), 140/161 with Ph− ALL achieved complete remission (86.9%; 91.6% ≤55 years, *P* = 0.0002), with complete MRD clearing in 68/109; 55 patients were assigned to maintenance chemotherapy, and 85 to HCT due to very high-risk characteristics (hyperleukocytosis, adverse genetics, early/mature T-precursor ALL, and MRD persistence). The 5-year relapse incidence was 36%, and the treatment-related mortality rate was 18%. Median overall and relapse-free survival were 7.4 and 6.2 years, with rates of 54 and 53% at 5 years, respectively, which were significantly better than those obtained with the historical protocol (*P* = 0.001 and *P* = 0.005, respectively), without significant differences between maintenance and HCT cohorts. In prognostic analysis, MRD negativity and age ≤55 years were the most favorable independent prognostic factors. A reduction in treatment toxicity and further improvements in the risk definitions and risk-oriented design are the focuses of this ongoing research.

## Introduction

Advances in the field of subset recognition, risk stratification, chemotherapy, targeted therapy, and immunotherapy have led to significant therapeutic progress in adult acute lymphoblastic leukemia (ALL) over the past 20 years^1,2^. In the frontline setting, outcomes were improved by the use of pediatric-inspired chemotherapy^3,4^, minimal residual disease (MRD) to optimize risk classification and guide treatments^5,6^, targeted therapy in Philadelphia chromosome-positive (Ph+) ALL^7^, and monoclonal antibody therapy in B-precursor ALL (B-ALL)^8^. Pediatric-based chemotherapy together with the assessment of postinduction MRD has been used in Ph− ALL as a primary risk classifier and indicator for risk-oriented allogeneic hematopoietic cell transplantation (HCT)^9–14^. While an MRD-driven postremission strategy is now uniformly recommended^15,16^ and widely adopted^17^, seminal MRD-oriented trials were conducted by the German Multicenter Group on Adult ALL (GMALL)^9–11^, the Northern Italy Leukemia Group (NILG)^18,19^, and the Programa Español de Tratamientos en Hematología (PETHEMA)^12^. Unlike the GMALL, which retained the indication for allogeneic HCT in MRD-negative (MRD_neg_) patients expressing high-risk (HR) features, both the NILG and PETHEMA adopted a chemotherapy-only approach in most MRD_neg_ patients irrespective of their clinical risk profile. Instead, MRD-positive (MRD_pos_) patients benefited from allogenic HCT in these and other studies^20–23^. Following the first MRD-based trial with an extensive analysis^18,19^, we focused on two major weaknesses of that study. First, complete remission (CR) induction and consolidation chemotherapy of the traditional adult type was less effective than modern pediatric-based regimens in HR, MRD_pos_, and T-precursor ALL (T-ALL) patients. The other reason for concern was the late timing of the MRD-guided decision for HCT (deferred until weeks 16–22 although an earlier week 10 MRD timepoint (TP) was already informative), which delayed the application of HCT in many MRD_pos_ and very high-risk (VHR) patients, increasing the risk of pretransplantation relapse. A new exploratory trial was designed adapting pediatric-type elements to the chemotherapy backbone and advancing the MRD risk definition for an early switch to allotransplantation in MRD_pos_ and VHR patients. Pediatric-type elements consisted of modified Berlin-Frankfurt-Münster (BFM) and lineage-targeted high-dose methotrexate (HD-MTX) courses, while the indication for allogeneic HCT was anticipated to week 10 after three chemotherapy blocks in all patients with MRD ≥ 10^−4^ and/or VHR characteristics. A randomized central nervous system (CNS) prophylaxis study was an integral part of the project^24^. Definitive trial results are reported and were compared with those from the previous study.

## Subjects and methods

### Patients and study design

**Fig. 1:**
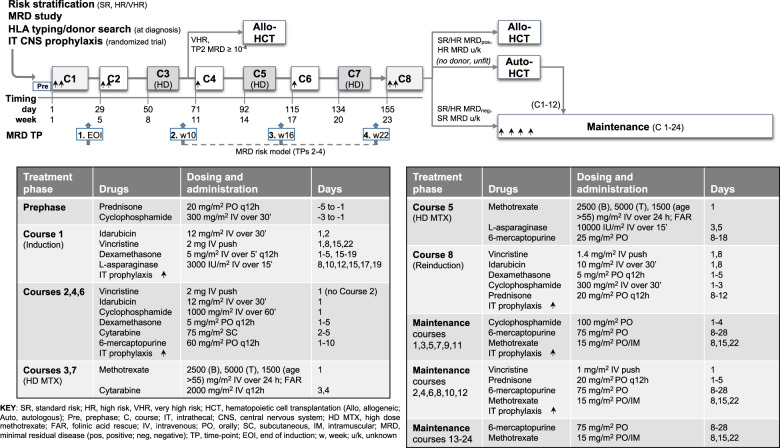
Induction–consolidation program and risk-oriented strategy. Study patients with Ph− ALL were risk stratified, randomized to IT CNS prophylaxis, and HLA-typed for the search for suitable HCT donors. Patients who achieved CR and were eligible for early allogeneic HCT displayed either a very HR (VHR) profile and/or TP2 MRD ≥ 10^−4^ or were HR without MRD results. Other SR and HR patients were allocated to maintenance chemotherapy when testing TP2-3 MRD negative (MRD_neg_) and to allogeneic HCT when testing MRD positive (MRD_pos_).

Eligible study patients had untreated ALL, were 18–65 years old, satisfied the enrollment criteria, and signed an informed consent form in accordance with the Helsinki Declaration of 1975, as revised in 2008. Protocol NILG ALL 10/07 was sponsored by the Ospedali Riuniti (currently Azienda Socio Sanitaria Territoriale Papa Giovanni XXIII) of Bergamo (Italy), approved by the Institutional Review Boards of the participating institutions and registered with ClinicalTrials.gov (Identifier NCT-00795756) (Fig. 1 and Supplements S1–2). The search for a family-related or unrelated HCT donor was activated at diagnosis.

### CR induction and consolidation chemotherapy

After 5-drug induction chemotherapy, postremission consolidation included three modified BFM blocks alternating with three lineage-targeted HD-MTX blocks associated with HD cytarabine and L-asparaginase. The modified BFM-type blocks included vincristine, dexamethasone, idarubicin (12 mg/m^2^), and cyclophosphamide (1000 mg/m^2^) on day 1, cytarabine and 6-mercaptopurine (Supplement S3, see the Toxicity section for details and related study amendments). Lineage-targeted HD-MTX, i.e., 2.5 and 5 g/m^2^ in B- and T-ALL, respectively, was patterned after studies conducted at St. Jude’s Hospital^25,26^ and applied to patients aged 18–55 years to ensure MTX through plasma levels of 33 and 65 µmol/l, respectively, followed by folinic acid rescue until the MTX plasma concentration was <0.25 µmol/l (Supplement S4). Patients with Ph+ ALL were treated with an imatinib-based deintensified chemotherapy regimen with a lower idarubicin dose and without L-asparaginase in induction, and with MTX 1.5 g/m^2^ and further dose reductions of cyclophosphamide, cytarabine, and 6-mercaptopurine in consolidation courses (Supplement S2).

### Integrated risk stratification

The standard risk (SR) subset was defined by a white blood cell (WBC) count <30 (x10^9^/l), a non-pro-B phenotype and a lack of the *BCR-ABL1* rearrangement in B-ALL, and a WBC count <100 plus the cortical CD1a+ phenotype in T-ALL. Postinduction risk stratification integrated ALL cytogenetics/genetics and MRD study results. MRD was assessed in the bone marrow at the end of CR induction (week 4/TP1) and during consolidation therapy at weeks 10, 16, and 22 (TPs 2–4) by means of sensitive (≥10^−4^) case-specific molecular probes. MRD results from TPs 2–4 were used for MRD-based risk definitions. HR patients were those not included in the SR or VHR group. VHR patients were those with a WBC count >100, adverse cytogenetics/genetics, and early/mature T-ALL, independent of MRD results. Adverse cytogenetics/genetics was defined as the t(4;11)/*KTM2A* rearrangement, abnormal 11q23, +8, −7, del6q, t(8;14), low hypodiploidy with 30–39 chromosomes, near-triploidy with 60–78 chromosomes, or a complex karyotype with ≥5 unrelated anomalies. In addition, all patients displaying a week 10/TP2 MRD ≥ 10^−4^ independent of their initial risk group were reclassified as VHR. The remaining SR and HR patients were risk restratified according to week 16–22/TP3–4 MRD results.

### Risk/MRD-oriented therapy

The final therapeutic elements were risk oriented (Fig. 1). All VHR patients, HR patients without MRD results, and SR/HR patients with TP2 MRD ≥ 10^−4^ (reclassified as VHR) were eligible for early HCT after course 3/TP2. SR/HR patients with TP2 MRD < 10^−4^ but positive TP3-4 MRD were eligible for the postconsolidation HCT group. Default choices dictated by clinical reasons or lack of suitable donors for allogeneic HCT-eligible patients were an autologous HCT after melphalan (200 mg/m^2^) conditioning plus maintenance for 12 months, or 2-year maintenance only when an autograft could not be performed. Instead, ALL SR/HR patients with TP2-3 MRD < 10^−4^ and TP4 MRD_neg_ and SR patients without MRD results were eligible to receive standard maintenance chemotherapy with daily 6-mercaptopurine and weekly MTX for 2 years plus monthly reinforcement alternating vincristine/prednisone and cyclophosphamide pulses during the first year.

### Trial objectives, definitions, and statistics

A major study objective was to determine whether the new risk-oriented strategy improved outcomes compared with the previous NILG trial. To ensure the validity of comparative analyses, baseline patient characteristics were compared between current and prior NILG trials. Of note, the current study patients were a median of 5 years older (*P* = 0.08) and were less likely affected by SR ALL (*P* = 0.04) than historical controls (Supplement S5). Data analysis was conducted according to the treatment intention and in discrete prognostic and treatment groups when appropriate using standard definitions of CR, early death, resistance, relapse, overall survival (OS), and relapse-free survival (RFS)^15,24^. Baseline patient characteristics are presented as numbers with percentages for categorical variables and medians with ranges for continuous variables. Differences in the induction response were assessed with Fisher’s exact test. Survival rates were estimated with the Kaplan–Meier method and compared using the log-rank test with 95% confidence intervals (CIs). The cumulative incidence of relapse (CIR) and treatment-related mortality (TRM) were estimated using the cumulative incidence function, considering death as a competing event for the CIR and relapse and death from other causes as competing events for TRM, using Gray’s test to assess differences between groups. The effects of prognostic factors on outcome were assessed using Cox models, comparing hazard ratios with 95% CIs. Allogeneic HCT was considered a time-dependent variable assessed with the Mantel-Byar test and graphically illustrated by Simon-Makuch plots^27,28^. *P* values were two-sided, with a 5% significance level. Statistics were performed with R software, version 3.5.0. The outcome of patients with Ph+ ALL was examined separately. These patients were normally allografted, as previously reported^29^.

## Results

### Patients, diagnosis, and trial disposition

**Fig. 2:**
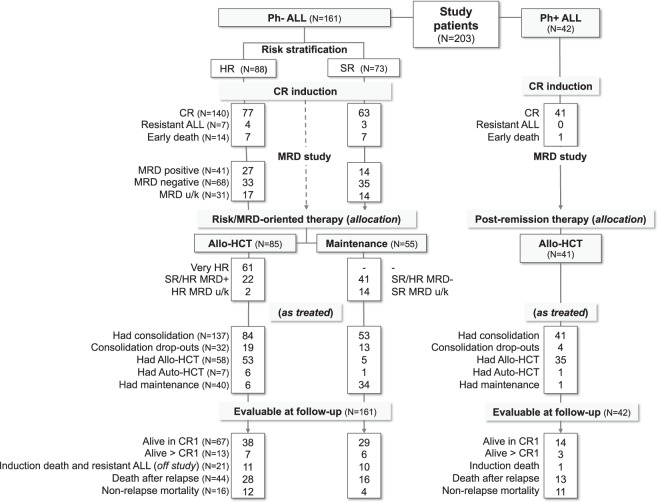
Flow chart of patient disposition and outcome. The study flow chart is shown according to the diagnosis of Ph− or Ph+ ALL, risk stratification, achievement of CR, and application of the risk-oriented strategy (*HR* high-risk, *SR* standard risk, *VHR* very HR, *CR* complete remission, *MRD* minimal residual disease, *HCT* hematopoietic cell transplantation). Notes: Consolidation dropouts in the Ph− ALL group: relapse (*n* = 23), toxicity and non-relapse mortality (*n* = 8), refusal (*n* = 1), and lost to follow-up (*n* = 1); early (*n* = 49) and postconsolidation (*n* = 9) HCT in the Ph− ALL group.

A total of 203 patients were enrolled between 2008 and 2012 (Table 1): 161 with Ph− ALL and 42 with Ph+ ALL. The incidence of T-ALL was 21.4%. The median patient age was 41 years and ranged from 17 to 67 years; 9 and 1 patients were aged 17–18 and >65 years, respectively. The percentage of bone marrow blast cells was usually >25%; six patients with 12–20% marrow blast cells were managed based on having ALL. At diagnosis, 73 (45.3%), 20 (12.4%), and 68 (42.2%) of the 161 Ph− ALL patients were classified as SR, HR and VHR, respectively. The VHR group included 36 patients with highly adverse genetics/cytogenetics, with 11 t(4;11)/*KMT2A* + ALLs, and four additional patients with abnormal 11q23. A total of 145 patients were randomized in the intrathecal (IT) CNS prophylaxis study; non-randomized patients received standard IT prophylaxis. The study flow chart with patient outcome is illustrated in Fig. 2.

**Table 1:** Demographics and other diagnostic characteristics of study patients.

	All patients (*n* = 203)	Ph− ALL (*n* = 161)		Ph+ ALL *n* = 42
		T-ALL (*n* = 44)	B-ALL (*n* = 117)	
Age (years), median (range)	41 (17–67)	38 (17–65)	42 (17–67)	43 (18–65)
≤55, *n* (%)	169 (83.3)	42 (95.5)	93 (79.5)	34 (81)
>55, *n* (%)	34 (16.7)	2 (4.5)	24 (20.5)	8 (19)
Gender (male), *n* (%)	114 (56.2)	28 (63.6)	66 (56.4)	20 (47.6)
Hemoglobin (g/dl), median (range)	9.8 (3.4–16.8)	11.1 (5.5–16.8)	9.6 (3.4–16)	10.8 (3.7–14.9)
WBC (10^9^/l), median (range)	11.3 (0.4–1021.4)	16.7 (1–281.2)	6.8 (0.4–1021.4)	19.9 (1.6–680)
>100, *n* (%)	32 (15.8)	10 (22.7)	15 (12.8)	7 (16.7)
BM blasts (%), median (range)^a^	90 (12–100)	90 (18–100)	90 (12–100)	90 (18–100)
PB blasts (%), median (range)	50 (0–100)	56 (0–100)	43 (0–98)	55.5 (2–99)
Platelets (10^9^/l), median (range)	58 (3–450)	70.5 (15–325)	57 (5–450)	43 (3–450)
Hepatomegaly, *n* (%)	36 (17.7)	7 (15.9)	23 (19.7)	6 (14.3)
Splenomegaly, *n* (%)	63 (31)	13 (29.5)	33 (28.2)	17 (40.5)
Lymphadenopathy, *n* (%)	37 (18.2)	19 (43.2)	13 (11.1)	5 (11.9)
Mediastina mass, *n* (%)	19 (9.4)	19 (43.2)	0 (0)	0 (0)
CNS involvement, *n* (%)	3 (1.5)	2 (4.5)	1 (0.9)	0 (0)
Immunophenotype, *n* (%)				
Pro-B	29 (14.4)	0 (0)	27 (23.3)	2 (4.8)
Common	97 (48)	0 (0)	62 (53.4)	35 (83.3)
Pre-B	32 (15.8)	0 (0)	27 (23.3)	5 (11.9)
Pro-T	6 (3)	6 (13.6)	0 (0)	N/A
Pre-T	13 (6.4)	13 (29.5)	0 (0)	
Cortical-T	21 (10.4)	21 (47.7)	0 (0)	
Mature-T	4 (2)	4 (9.1)	0 (0)	
Cytogenetics/genetics, *n* (%)				
Normal	77 (37.9)	26 (59.1)	51 (43.6)	N/A
Adverse	78 (38.4)	9 (20.5)	27 (23.1)	42 (100)
t(9;22)/*BCR-ABL1*	42 (20.7)	0 (0)	0 (0)	42 (100)
t(4;11)/*KMT2A-AFF4*	11 (5.4)	0 (0)	11 (9.4)	N/A
Other^b^	25 (12.3)	9 (20.5)	16 (13.7)	
Non-adverse	25 (12.3)	4 (9.1)	21 (17.9)	
t(1;19)/*E2A-PBX1*	2 (1)	1 (2.3)	1 (0.9)	
Hyperdiploid	5 (2.5)	0 (0)	5 (4.3)	
Other	18 (8.9)	3 (6.8)	15 (12.8)	
Not known	23 (11.3)	5 (11.4)	18 (15.4)	
Risk stratification^c^, *n* (%)				N/A
Standard-risk	–	11 (25)	62 (52.9)	
High-risk	–	0 (0)	20 (17)	
Very high-risk	–	33 (75)	35 (29.9)	

*Ph* Philadelphia chromosome and/or *BCR-ABL1* rearrangement, *ALL* acute lymphoblastic leukemia, *WBC* white blood cells, *BM* bone marrow, *PB* peripheral blood, *CNS* central nervous system, *N/A* not applicable/available (outside study project).

^a^Including 6 patients with BM blast cell content between 12 and 20%: 2 and 4 had a diagnosis of T- and B-precursor lymphoblastic leukemia/lymphoma, respectively (1 had Ph+ ALL with 18% BM blasts); 4 of these 6 patients also had detectable PB blasts (2–10%).

^b^Other adverse abnormalities included abn 11q23 (*n* = 4), −7 (*n* = 5), t(8;14) *(n* = 2), del(6q) (*n* = 5), near triploid (*n* = 2), +8 (*n* = 4), complex karyotype with five or more abnormalities (*n* = 12).

^c^Only patients with Ph− ALL (*n* = 161).

### Achievement of CR

A total of 181 patients achieved CR (89.2%): 41 with Ph+ ALL (97.6%) and 140 with Ph− ALL (86.9%). The CR rate was 97.7% in T-ALL patients as opposed to 82.9% in Ph− B-ALL patients, mainly because of the high early mortality in patients aged >55 years (Table 2 and Toxicity section below). Remission induction was fast: 137 patients achieved CR at course 1, and three at course 2. Seven unresponsive patients were excluded from the study (refractory T-ALL 2.2% and 5.1% B-ALL).

**Table 2:** Main outcome results in 161 patients with Ph− ALL also according to patient age (≤55 vs. >55 years) and ALL subset (B-ALL vs. T-ALL) (95% CI in brackets for time-dependent variables).

	All patients (*n* = 161)	Age groups			ALL subsets		
		≤55 years (*n* = 135)	>55 years (*n* = 26)	*P* value^a^	B-ALL (*n* = 117)	T-ALL (*n* = 44)	*P* value^a^
CR induction							
CR, no. (%)	140 (86.9)	124 (91.9)	16 (61.5)	0.0002	97 (82.9)	43 (97.7)	0.02
NR, no. (%)	7 (4.3)	7 (5.2)	0 (0)	0.60	6 (5.1)	1 (2.3)	0.67
ED, no. (%)	14 (8.6)	4 (2.9)	10 (38.5)	<0.0001	14 (12.0)	0 (0)	0.01
Treatment-related mortality^b^							
No. (%)	28 (17.4)	14 (10.4)	14 (53.8)	<0.0001	25 (21.4)	3 (6.8)	0.02
5–10 years (%)	18 (12–24)	10 (6–16)	55 (33–72)		22 (15–29)	7 (2–17)	
Cumulative incidence of relapse^c^							
No. (%)	54 (33.5)	49 (36.3)	5 (19.2)	0.70	40 (24.2)	14 (31.8)	0.24
5 years (%)	36 (28–44)	36 (28–45)	32 (11–56)		38 (28–48)	30 (17–44)	
10 years (%)	40 (32–49)	41 (32–50)	N/A		44 (33–54)	33 (19–48)	
Relapse-free survival^c^							
Median (years)	6.3	7.2	2.0	0.06	4.9	N/A	0.16
5 years (%)	53 (45–62)	56 (47–65)	28 (12–64)		49 (40–60)	60 (47–77)	
10 years (%)	45 (37–55)	48 (39–58)	N/A		43 (33–54)	50 (35–73)	
CR duration^c^							
Median (years)	N/A	N/A	N/A	0.96	N/A	N/A	0.19
5 years (%)	62 (54–71)	62 (54–71)	61 (39–96)		59 (49–70)	68 (55–84)	
10 years (%)	56 (48–66)	56 (48–67)	N/A		52 (42–65)	64 (51–82)	
Event-free survival^d^							
Median (years)	3.4	5.4	0.5	<0.0001	1.9	8.9	0.02
5 years (%)	46 (39–55)	52 (44–61)	17 (7–41)		42 (33–52)	59 (46–75)	
10 years (%)	39 (32–49)	44 (35–54)	N/A		35 (27–46)	49 (34–71)	
Overall survival							
Median (years)	7.4	N/A	0.6	<0.0001	3.8	N/A	0.002
5 years (%)	52 (45–62)	60 (52–69)	21 (9–45)		47 (38–57)	73 (61–87)	
10 years (%)	46 (38–56)	52 (44–63)	N/A		40 (32–51)	63 (48–84)	

*ALL* acute lymphoblastic leukemia, *CI* confidence interval, *CR* complete remission, *NR* non-responsive, *ED* early death, *N/A* not achieved.

^a^Fisher test, Log-rank test, or Gray test, as appropriate.

^b^Cumulative: sum of CR induction mortality and non-relapse mortality in CR patients (with censoring of 2 patients who died of an illness unrelated to ALL and its management).

^c^Calculated on 140 CR patients, including 4 patients with MRD relapse in relapse incidence and relapse-free survival analysis, with censoring of treatment-related deaths and secondary myeloid malignancies (*n* = 3) in CR duration analysis.

^d^Calculated on all study patients from diagnosis to induction death/resistance/recurrence/death in CR or last follow-up, whichever occurred first, with censoring of secondary AML/MDS (*n* = 3).

### MRD results and risk stratification

A sensitive molecular probe was available for 112 patients who achieved CR (80%); of these, 109 were ultimately classified as MRD_pos_ (*n* = 41, 37.6%) or MRD_neg_ (*n* = 68, 62.4%), 102 exactly according to the study risk model and seven missing a single MRD TP but otherwise displaying consistent MRD study results (Table 3). The correlation between the end-of-induction morphological marrow CR and TP1 MRD revealed a deep MRD response (<10^−4^) in 52.1% of patients (36.9% MRD negative): 62.5% in the SR group (45% MRD negative) and 44.2% in the HR/VHR group (30.7% MRD negative). At the most critical MRD TP2, MRD was <10^−4^ in 70.8% of patients (60.4% MRD negative): 80.8% in the SR group (70.2% MRD negative) and 62.7% in the HR/VHR group (52.5% MRD negative) (*P* = 0.07 and *P* = 0.09 for MRD < 10^−4^ and negative, respectively). Comparable data were observed in SR patients at TP3 (67.6% MRD negative) and TP4 (76.3% MRD negative) and in HR/VHR patients (72.2 and 60% MRD negative, respectively), although in small patient groups due to increasing study losses and the early HCT policy (Supplement S6). In the final risk model, 14 (22.2%) SR patients and 6 (37.5%) HR patients were risk restratified as MRD_pos_ and MRD_neg_, respectively; 27 (58.6%) VHR patients were confirmed to be MRD_neg_, which did not affect their treatment design. The lack of a case-specific MRD probe(s) and/or bad marrow sampling prevented MRD analysis in 31 patients.

**Table 3:** Combined risk stratification for assignment to risk-oriented therapy in Ph− ALL (*n* = 140).

	All patients (*n* = 140)	T-ALL			B-ALL			
		All (*n* = 43)	SR (*n* = 11)	VHR (*n* = 32)	All (*n* = 97)	SR (*n* = 52)	HR (*n* = 16)	VHR (*n* = 29)
End of induction MRD (TP1), *n* (%)								
Evaluable	92	33 (76.7)	8 (72.7)	25 (78.1)	59 (60.8)	32 (61.5)	9 (56.3)	18 (62.1)
Negative	34 (37.0)	15 (45.5)	5 (62.5)	10 (40.0)	19 (32.2)	13 (40.6)	1 (11.1)	5 (27.8)
<10^–4^	14 (15.2)	4 (12.1)	1 (12.5)	3 (12.0)	10 (16.9)	6 (18.8)	1 (11.1)	3 (16.7)
≥10^–4^	44 (47.8)	14 (42.4)	2 (25.0)	12 (48.0)	30 (50.8)	13 (40.6)	7 (77.8)	10 (55.6)
Early consolidation MRD (TP2), *n* (%)								
Evaluable	106	36 (83.7)	10 (90.9)	26 (81.3)	70 (72.2)	37 (71.2)	14 (87.5)	19 (65.5)
Negative	64 (60.4)	22 (61.1)	8 (80.0)	14 (53.8)	42 (60.0)	25 (67.6)	6 (42.9)	11 (57.9)
<10^–4^	11 (10.4)	5 (13.9)	1 (10.0)	4 (15.4)	6 (8.6)	4 (10.8)	2 (14.3)	0 (0.0)
≥10^–4^	31 (29.2)	9 (25.0)	1 (10.0)	8 (30.8)	22 (31.4)	8 (21.6)	6 (42.9)	8 (42.1)
MRD risk model^a^, *n* (%)								
Evaluable	109 (77.9)	36 (83.7)	10 (90.9)	26 (81.3)	73 (75.3)	39 (75.0)	14 (87.5)	20 (69.0)
MRD_pos_	41 (37.6)	10 (27.8)	2 (20.0)	8 (30.8)	31 (42.5)	12 (30.8)	8 (57.1)	11 (55.0)
MRD_neg_	68 (62.4)	26 (72.2)	8 (80.0)	18 (69.2)	42 (57.5)	27 (69.2)	6 (42.9)	9 (45.0)
Allocation cohort, *n* (%)								
Maintenance	55 (39.3)	9 (20.9)	9 (81.8)	–	46 (47.4)	40 (76.9)	6 (37.5)	–
SR MRD_neg_	35 (63.6)	8 (88.9)	8 (88.9)	–	27 (58.7)	27 (67.5)	–	–
SR MRD_u/k_	14 (25.5)	1 (11.1)	1 (11.1)	–	13 (28.3)	13 (32.5)	–	–
HR MRD_neg_	6 (10.9)	–	–	–	6 (13.0)	–	6 (100.0)	–
Allogeneic HCT	85 (60.7)	34 (79.1)	2 (18.2)	32 (100.0)	51 (52.6)	12 (23.1)	10 (62.5)	29 (100.0)
VHR	61 (71.8)	32 (94.1)	–	32 (100.0)	29 (56.9)	–	–	29 (100.0)
HR MRD_pos_	8 (9.4)	–	–	–	8 (15.7)	–	8 (80.0)	–
HR MRD_u/k_	2 (2.4)	–	–	–	2 (3.9)	–	2 (20.0)	–
SR MRD_pos_	14 (16.5)	2 (5.9)	2 (100.0)	–	12 (23.5)	12 (100.0)	–	–

*ALL* acute lymphoblastic leukemia, *SR* standard risk, *HR* high-risk, *VHR* very high-risk, *TP* timepoint, *MRD* minimal residual disease, *neg* negative, *pos* positive, *u/k* unknown, *HCT* hematopoietic cell transplantation. MRD-based risk classification was available for 109 patients. Details of MRD analysis are shown for TP1 (end of induction) and TP2 according to ALL subset and clinical risk stratification (SR, HR, VHR). TP3 and TP4 MRD results are reported in supplemental file. MRD study results were obtained before any HCT.

^a^As based on TP2, TP3, and TP4 MRD analysis.

### Risk/MRD-oriented therapy

Upon completion of the risk stratification process, all patients who achieved CR were assigned to receive either allogeneic HCT (*n* = 85, 60.7%) or standard maintenance chemotherapy (*n* = 55, 39.3%) (Fig. 2 and Table 3). Seventy-eight patients were allocated to undergo early HCT (61 VHR, 2 HR without MRD results, and 15 SR/HR TP2 MRD_pos_), and 7 TP3-4 MRD_pos_ patients were allocated to receive postconsolidation HCT. In this cohort, 53 of the 78 patients actually underwent allogeneic HCT, and six underwent autologous HCT, for a global transplantation rate of 69.4%. Fifty-five patients (41 SR/HR MRD_neg_ and 14 SR without MRD results) were allocated to receive maintenance chemotherapy. In this cohort, six patients were switched to HCT because of poor chemotherapy tolerance or medical decisions. Altogether, 35 patients were excluded from HCT or maintenance due to ALL recurrence (*n* = 25), treatment toxicity (*n* = 8), and refusal and loss to follow-up (*n* = 2).

### Overall study results

**Fig. 3:**
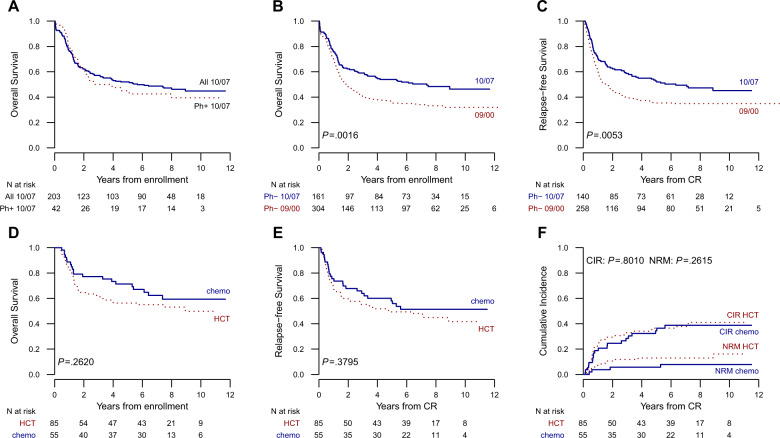
Main study results. Kaplan–Meier graphs illustrating the overall survival of all 203 study patients and 42 patients with Ph+ ALL (**A**) and of 161 patients with Ph− ALL compared with prior study results (**B**), relapse-free survival of 140 CR patients with Ph− ALL compared with prior study results (**C**), overall survival (**D**), relapse-free survival (**E**), and cumulative incidence of relapse (CIR) and non-relapse mortality (NRM) in CR patients with Ph− ALL assigned to either chemotherapy (chemo) or allogeneic HCT (**F**).

With a median follow-up of 7.8 years (0.3–11.7 years), the median OS time for all 203 study patients was 5.7 years, with a projected 5-year rate of 52 and 45% in Ph+ ALL patients (Fig. 3A and Supplement S7). Treatment outcomes of patients with Ph− ALL are summarized in Table 2. The median OS time was 7.4 years, with 5- and 10-year estimates of 54 and 46%, respectively, which were significantly better than those obtained in the prior NILG study (Fig. 3B). Eighty patients survived 5+ years: 67 in CR1 and 13 beyond CR1. Eighty-one patients died: 77 because of ALL and/or therapy-related complications (47.8%), two because of a secondary myeloid neoplasm, and two because of a non-hematologic cancer and liver cirrhosis. Cumulative TRM affected 14 patients in induction therapy and 14 during postremission therapy (17.4%). The median RFS time was 6.3 years, with 5- and 10-year estimates of 53 and 45%, respectively, which were significantly better than those reported in prior study (Fig. 3C). Fifty patients who achieved CR relapsed clinically (35.7%), four had molecular relapse managed as recurrent ALL (2.8%), and three developed a secondary myeloid malignancy. When compared with the historical patient series, the 5-year CIR rate was significantly reduced across all clinical risk groups (Supplement S8). Relapse occurred within 2 years from CR in 38 patients (70.4%), between 2 and 5 years in 11, and beyond 5 years in five. The sites of recurrence were the bone marrow (*n* = 44), CNS (*n* = 2), marrow plus CNS (*n* = 4), and other extramedullary sites (*n* = 4). The median survival time from relapse was 0.6 years, with estimated rates of 29 and 20% at 2 and 5 years, respectively.

### Treatment results according to risk-oriented therapy

Median survival was not reached in the chemotherapy allocation cohort, with a projected 5-year rate of 71%, and was 8.9 years in the HCT allocation cohort, with a 5-year projection of 56% (Table 4 and Fig. 3D). Corresponding RFS figures were not reached and 4.8 years (median time), 58 and 49% at 5 years, respectively (Fig. 3E). These results were not significantly different. The risks of relapse and non-relapse mortality were also similar: 34 vs. 37% and 6 vs. 14% at 5 years, respectively (Fig. 3F). After censoring treatment-related deaths, the CR duration estimates were 60 and 64% in the two allocation cohorts, respectively.

**Table 4:** Outcome results and univariate prognostic analysis in different risk and treatment subsets in Ph− ALL (95% CI within brackets).

Study parameter	OS (*n* = 161)^a^				CIR and RFS (*N* = 140)						
					CIR				RFS		
	No.	5-year (%)	HR	*P*	No.	5-year (%)	HR	*P*	5-year (%)	HR	*P*
Age (years)											
≤55	135	60 (52–69)	1		124	36 (28–45)	1		56 (47–65)	1	
>55	26	21 (10–45)	3.4 (2.06–5.61)	<0.0001	16	32 (11–56)	0.81 (0.31–2.07)	0.66	28 (12–64)	1.83 (0.96–3.48)	0.07
Gender											
Female	67	58 (48–72)	1		61	24 (14–35)	1		59 (48–73)	1	
Male	94	51 (42–62)	1.22 (0.78–1.93)	0.38	79	45 (33–55)	1.97 (1.09–3.56)	0.025	48 (38–60)	1.34 (0.83–2.18)	0.23
WBC (10^9^/l)											
<30	115	56 (48–66)	1		99	35 (26–45)	1		55 (46–66)	1	
30–100	21	47 (30–74)	1.49 (0.82–2.73)	0.19	18	33 (13–55)	1.02 (0.46–2.27)	0.96	44 (27–74)	1.45 (0.76–2.8)	0.26
>100	25	48 (32–72)	1.09 (0.6–2)	0.77	23	39 (19–59)	1.13 (0.51–2.49)	0.76	48 (31–73)	1.26 (0.67–2.37)	0.47
BM blasts (%)											
≤50	21	57 (39–83)	1		17	18 (4–39)	1		71 (52–96)	1	
>50	140	54 (46–63)	0.99 (0.51–1.91)	0.96	123	38 (29–47)	2.62 (0.8–8.62)	0.11	50 (42–60)	1.66 (0.72–3.83)	0.23
Hepato-splenomegaly											
No	108	58 (49–68)	1		93	30 (21–39)	1		60 (50–70)	1	
Yes	53	46 (34–62)	1.46 (0.93–2.28)	0.10	47	48 (33–62)	1.71 (1–2.91)	0.051	39 (27–56)	1.84 (1.15–2.94)	0.01
Mediastinal mass											
No	142	50 (42–59)	1		121	39 (30–48)	1		49 (41–59)	1	
Yes	19	84 (69–100)	0.28 (0.1–0.78)	0.01	19	16 (4–36)	0.32 (0.09–1.06)	0.062	74 (56–96)	0.39 (0.16–0.97)	0.04
CNS											
No	158	54 (47–63)	1		137	35 (27–43)	1		53 (45–62)	1	
Yes	3	33 (7–100)	1.28 (0.31–5.2)	0.73	3	67 (0–97)	1.91 (0.58–6.33)	0.29	33 (7–100)	1.35 (0.33–5.5)	0.68
Immunophenotype											
B-ALL	117	47 (38–57)	1		97	38 (28–48)	1		49 (40–60)	1	
T-ALL	44	73 (61–87)	0.42 (0.24–0.75)	0.003	43	30 (17–44)	0.7 (0.38–1.28)	0.25	60 (47–77)	0.69 (0.41–1.16)	0.16
B-ALL											
Pro-B	27	37 (23–61)	1		22	50 (27–69)	1		41 (25–68)	1	
“Common”	62	41 (30–56)	0.90 (0.52–1.55)	0.70	51	39 (25–53)	0.78 (0.39–1.57)	0.49	42 (30–59)	1.05 (0.56–1.98)	0.87
Pre-B	27	70 (55–90)	0.32 (0.14–0.73)	0.007	23	22 (8–41)	0.31 (0.11–0.88)	0.03	74 (58–94)	0.31 (0.12–0.80)	0.02
T-ALL											
Cortical-T	21	76 (60–97)	1		21	29 (11–49)	1		62 (44–87)		
Non-cortical-T	23	69 (53–91)	1.27 (0.44–3.67)	0.66	22	32 (14–52)	0.87 (0.31–2.43)	0.79	59 (42–84)	1.00 (0.40–2.47)	1.00
Cytogenetics/genetics											
Normal	77	59 (49–71)	1		71	28 (18–39)	1		55 (44–68)	1	
Non-adverse	36	61 (47–79)	0.89 (0.49–1.63)	0.70	30	30 (15–47)	0.85 (0.4–1.81)	0.67	63 (48–83)	0.63 (0.32–1.23)	0.17
Adverse	25	44 (28–68)	1.57 (0.86–2.86)	0.14	21	52 (29–72)	2.04 (1.02–4.05)	0.04	43 (26–70)	1.46 (0.79–2.71)	0.23
t(4;11)/*KMT2A*+											
No	146	53 (46–62)	1		126	37 (28–45)	1		51 (43–61)	1	
Yes	15	60 (40–91)	0.71 (0.31–1.63)	0.41	14	29 (8–53)	0.67 (0.23–1.89)	0.45	64 (44–95)	0.59 (0.24–1.48)	0.26
Risk stratification											
SR	73	58 (47–70)	1		63	31 (20–43)	1		55 (44–69)	1	
HR/VHR	88	51 (41–63)	1.13 (0.73–1.76)	0.58	77	39 (28–50)	1.21 (0.71–2.07)	0.48	51 (41–63)	1.09 (0.68–1.73)	0.72
MRD											
Negative	68	78 (69–88)	1		68	24 (14–34)	1		66 (56–78)	1	
Positive	41	34 (22–52)	3.57 (2–6.37)	<0.0001	41	54 (37–68)	3.06 (1.68–5.59)	0.0003	29 (18–47)	3.08 (1.82–5.21)	<0.0001
TP1 and TP2 MRD											
Both <10^–4^	42	76 (64–90)	1		42	14 (6–27)	1		71 (59–86)	1	
Discordant	21	71 (54–94)	1.37 (0.56–3.36)	0.49	21	48 (25–67)	3.59 (1.51–8.55)	0.004	52 (35–79)	1.71 (0.8–3.66)	0.167
Both ≥10^–4^	23	39 (24–65)	3.23 (1.51–6.92)	0.003	23	43 (22–63)	2.75 (1.07–7.08)	0.04	35 (20–61)	2.53 (1.23–5.18)	0.011
MRD and risk stratification											
MRD_neg_ SR	35	83 (71–96)	1		35	23 (11–38)	1		68 (84–56)	1	
MRD_neg_ HR/VHR	33	72 (58–90)	1.07 (0.44–2.56)	0.89	33	24 (11–40)	1.06 (0.43–2.65)	0.89	63 (49–82)	1.06 (0.49–2.29)	0.88
MRD_pos_ SR	14	36 (18–72)	1		14	29 (8–53)	1		36 (18–72)	1	
MRD_pos_ HR/VHR	27	33 (20–57)	1.25 (0.56–2.76)	0.58	27	67 (45–81)	2.20 (0.94–5.17)	0.07	26 (14–49)	1.24 (0.59–2.59)	0.57
Treatment allocation											
Chemotherapy	55	71 (60–85)	1		55	34 (22–47)	1		58 (46–73)	1	
HCT	85	56 (47–68)	1.36 (0.79–2.33)	0.26	85	37 (26–47)	1.08 (0.62–1.86)	0.79	49 (40–61)	1.24 (0.76–2.02)	0.38
HCT allocation cohort^b^											
HCT+	59	66 (51–87)	1		59	21 (12–37)	1		61 (49–76)	1	
HCT−	26	36 (21–60)	3.36 (1.64–6.89)	0.0009	26	65 (48–88)	4.48 (2.01–10.00)	0.0002	29 (14–61)	2.41 (1.19–4.88)	0.01
HR/VHR risk class and HCT^b^											
HCT+	45	69 (53–90)	1		45	21 (12–38)	1		67 (54–82)	1	
HCT−	18	39 (21–71)	3.27 (1.38–7.73)	0.007	18	69 (48–99)	4.73 (1.87–11.98)	0.001	28 (12–68)	3.08 (1.33–7.13)	0.008
MRD_pos_ and HCT^b^											
HCT+	23	35 (15–86)	1		23	25 (12–50)	1		43 (26–70)	1	
HCT−	18	14 (4–49)	2.67 (1.14–6.24)	0.02	18	85 (66–100)	4.34 (1.53–12.28)	0.009	12 (2–71)	2.21 (0.88–5.54)	0.09

*OS* overall survival, *CIR* cumulative incidence of relapse, *RFS* relapse-free survival, *CI* confidence intervals, *HR* hazard ratio, *WBC* white blood cell, *BM* bone marrow, *CNS* central nervous system, *ALL* acute lymphoblastic leukemia, *SR* standard risk, *HR* high-risk, *VHR* very HR, *MRD* minimal residual disease, *neg* negative, *pos* positive, *u/k* unknown, *HCT* hematopoietic cell transplantation, *N/A* not achieved. OS analysis performed in 161 of the total patients or 140 CR patients (or fewer, when applicable [cytogenetics, MRD study, and HCT]) to assess interactions between risk class, MRD subset, postremission therapy allocation and allogeneic HCT.

^a^Additional prognostic analysis on 140 CR patients or less, depending on available data, as indicated in the table.

^b^HCT as time-dependent variable (HCT allocation criteria as per study design: 1. VHR regardless of MRD status, 2. HR MRD_u/k_, 3. SR/HR MRD_pos_).

### Prognostic analysis

**Fig. 4:**
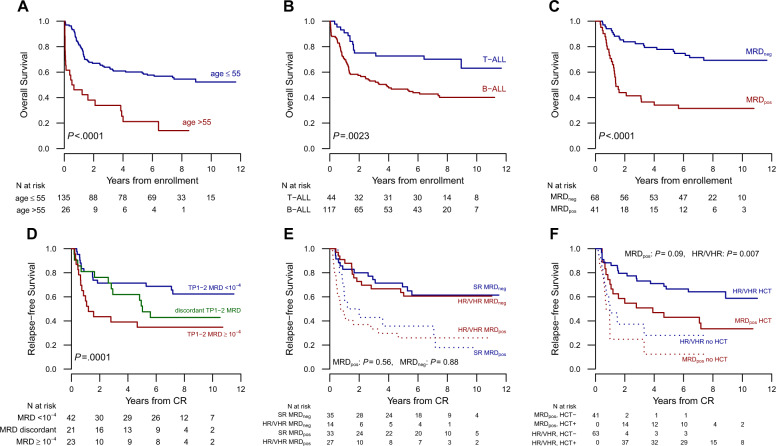
Study results according to prognostic characteristics and treatment allocation. Kaplan–Meier graphs illustrating the overall survival of study patients with Ph− ALL according to patient age ≤55 vs. >55 years (**A**), diagnosis of B-ALL vs. T-ALL (**B**), achievement of the MRD_neg_ vs. MRD_pos_ status (**C**), relapse-free survival according to the MRD_neg_ status achieved at TP1 and confirmed at TP2 vs. not (**D**), MRD status in SR vs. HR/VHR groups (**E**), and time-dependent application of allogeneic HCT (yes vs. no) by risk class and the MRD status (**F**).

In the univariate analysis (Table 4), outcome was significantly improved in patients aged 55 years and younger (CR and OS, *P* < 0.0001; RFS, *P* = 0.06; Fig. 4A), in female patients (CIR, *P* = 0.03), in those with T-ALL due to the high CR rate (RFS, *P* = 0.04; OS, *P* = 0.003; Fig. 4B), in those without hepato-splenomegaly (RFS, *P* = 0.01), and especially in those who achieved an MRD_neg_ status (CIR, *P* = 0.0003; RFS and OS, *P* < 0.0001; Fig. 4C). Notably, an end-of-induction TP1 MRD_neg_ status maintained at TP2 predicted a low relapse risk (14%) and prolonged RFS (Fig. 4D). The favorable prognostic effect on the MRD_neg_ status was confirmed across all risk subsets (Fig. 4E). The risk of relapse was increased by HR cytogenetics (CIR, *P* = 0.04) but not by t(4;11)/*KMT2A* + ALL considered alone, a high WBC count or predetermined HR phenotypes. In the multivariable analysis, age >55 years and the MRD_pos_ status retained a strongly negative prognostic effect on OS (HR 3.40 [95% CI, 1.36–8.54] and HR 3.83 [95% CI, 1.90–7.69], *P* = 0.009 and *P* = 0.002) and RFS (HR 3.52 [95% CI, 1.41–8.76] and 3.55 (95% CI, 1.87–6.75], *P* = 0.007 and *P* = 0.0001), while the risk of relapse was significantly affected by MRD only (HR 3.69 [95% CI, 1.66–8.19], *P* = 0.001).

### Role of allogeneic HCT in the HR/VHR and MRD_pos_ subsets

The prognostic effect of allogeneic HCT was examined in a time-dependent manner in the two risk subsets independently assigned to this treatment (VHR and HR without MRD results or MRD_pos_). The prognostic benefit conferred by an allograft in either condition appeared substantial (Table 4), since the incidence of relapse following HCT was 21% in HR/VHR patients and 25% in MRD_pos_ patients compared to 69 and 85% without HCT (*P* = 0.0009 and *P* = 0.002), respectively, and was associated with an improved clinical outcome in the HCT group (Fig. 4F).

### Treatment-related toxicity

The risk of induction mortality was high in patients aged >55 years with Ph− ALL (Table 2). Thirteen of the 14 induction-related deaths were related to pancytopenia infectious complications, with a documented etiology in eight instances (6 Gram–, 1 Gram+ bacteria, and 1 *Aspergillus* spp.), and one to intracranial hemorrhage. The single induction failure in Ph+ ALL was caused by *Legionella* spp. pneumonia. Toxicities associated with BFM-like and HD-MTX courses were examined (Supplement S9). At the start of the study, severe myelotoxicity associated with modified BFM-like consolidation caused three pancytopenia-related deaths among patients aged >55 years. Following a study amendment that shortened the cytarabine and 6-mercaptopurine phase from 8 and 14 days to 4 and 10 days, respectively, no further death was reported. Toxicity associated with lineage-targeted HD-MTX courses was less severe, with few serious adverse events (CTC grade >2). All events were reversible and did not hamper the indication for associated or subsequent chemotherapy. The MTX infusion reached the intended drug plasma levels in the majority of the patients treated (Supplement S10).

## Discussion

The long-term, very mature results of the current trial documented significant therapeutic progress in adult Ph− ALL compared with the previous NILG study^18,19^. The pediatric-inspired chemotherapy regimen used along with an early allotransplantation policy oriented by risk class and MRD yielded a high CR rate and maintained a relapse incidence of approximately 35% at 5 years, allowing a cure in approximately half of the study patients. These figures were achieved in a series with a median patient age of 40 years (range 18–65 years), which ranks high among adult ALL studies and puts one-half of the study patients outside the favorable adolescent and young adult (AYA) category^30–34^.

The age issue is critical in ALL therapy^35^ and becomes of special concern in older patients who receive intensive pediatric-type regimens, whose associated toxicity can offset the therapeutic benefit consistently reported in AYAs <35–45 years. Moreover, older adults incur higher transplant-related mortality, which limits further treatment efficacy, and are more likely to express poor-risk ALL genetics and cytogenetics^36,37^, which are predictive of an inferior outcome. Fifty-five years was set as the age-related prognostic cut-off, and HCT-related mortality was taken into account, thereby increasing the cumulative TRM in our study from 8 and 13% in patients aged ≤55 years who did not undergo and underwent HCT, respectively, to 52% in those older than 55 years. The Group for Research in Adult ALL (GRAALL) reported in two consecutive trials a cumulative incidence of induction and CR mortality of 41.5 and 29% above 45 and 55 years of age, respectively^38,39^, and even in AYAs aged 18–45 years, this risk was estimated to be between 6.7 and 12%^34,40^. Because induction mortality was a major drawback in patients aged >55 years (*P* < 0.0001), the cyclophosphamide and anthracycline doses were attenuated in the successor trial, improving both CR and early survival figures (CR 87.1% and 1-year OS 73.2%, *P* = 0.08)^41^. Apart from age-dependent hazards, the 5-year OS and DFS rates were estimated at 60 and 55%, respectively, in patients aged ≤55 years, reflecting the curative potential of the new strategy given the long follow-up extension.

The concept of pediatric-type chemotherapy here embraced the BFM-like and lineage-targeted HD-MTX consolidation courses, rotating six times after CR. The BFM-derived schedule was modified by using a single cyclophosphamide dose at 1000 mg/m^2^ and adding vincristine, dexamethasone, and idarubicin, the latter owing to prior experience with dose-intensive anthracyclines in SR B-ALL^42^. These modifications caused severe myelotoxicity, with some therapy-related deaths at the start of the study, requiring an amendment that shortened the exposure to cytarabine and 6-mercaptopurine. Lineage-targeted HD-MTX was used at 2.5 g/m^2^ in B-ALL and 5 g/m^2^ in T-ALL^25,26^ to ensure optimal drug plasma concentrations of approximately 33 and 65 µmol/l, respectively. Although individual MTX plasma concentrations were not assessed to adjust the drug infusion rate as in the original St. Jude’s Hospital study XV^43^, the desired drug level was recorded in most instances, and the treatment proved feasible with few reversible toxic side effects even in association with HD cytarabine (2 g/m^2^) or L-asparaginase; this HD-MTX schedule may therefore deserve further investigation in adult ALL, as suggested by other studies^44–46^. Differing from other recent AYA and adult trials^33,34,47,48^, pegylated-asparaginase (Peg-ASP) was not used, and instead, only low- and standard-dose *E. coli* L-asparaginase was administered during induction and consolidation; the question of whether Peg-ASP could enhance the therapeutic power of this regimen was addressed in a subsequent trial with favorable early results^41^.

A typical feature of the study was an early allocation to allogeneic HCT in patients with suitable risk characteristics. With an expected CR rate of approximately 90%, the search for a related/unrelated HCT donor was activated at diagnosis to facilitate early access to the procedure. The final decision to proceed with HCT in CR1 was made upon the joint assessment of the patient risk profile and postinduction MRD, and eventually, it concerned more than one-half of all patients who achieved CR because of their VHR characteristics (WBC count >100, highly adverse cytogenetics/genetics, and HR T-cell subsets), historically associated with a poor outcome, and/or early MRD resistance (MRD ≥ 10^−4^). With the molecular MRD results available in 77% of patients who achieved CR and the decision to transplant all HCT-eligible patients after chemotherapy course 3, 62% of all eligible patients actually underwent allogeneic HCT, which represents an improvement over the historical figure (43%)^19^, although many were still excluded from an allograft because of pretransplantation relapse, as documented in the time-dependent analyses. Nonetheless, outcome was similar among the chemotherapy and HCT allocation cohorts, purporting a good outcome with an allograft for patients with a poor risk profile^11,17,49,50^ despite the high net non-relapse mortality expected with HCT^22,50^.

Considering the MRD-based and therapy-oriented risk classification, accumulating evidence suggests that poor-risk cytogenetics/genetics predict relapse independent of the MRD risk classification^21,51^. An integrated prognostic index involving ALL genetics, WBC count, and MRD, recently tested in an adult United Kingdom (UK) ALL trial^23^, predicted, with considerable accuracy, either posttransplantation relapse after myeloablative (relapse risk 52%) and reduced-intensity (relapse risk 49%) conditioning or excellent survival after chemotherapy only (88% at 3 years, relapse risk 12%). Our mixed risk classification system essentially reflected the same variables (plus an adverse T-cell phenotype), albeit with dichotomous rather than mathematical risk modeling. With these parameters, the relapse rate was affected by risk class and was the highest in patients displaying MRD resistance (overall relapse 54%, 23% after allogeneic HCT) and the lowest in patients with an end-of-induction and early consolidation MRD < 10^−4^ (relapse 14% at 5 years). Although a weakness of our study was the relatively small number of patients in some risk subsets, the results were consistent with the general experience of the inferior feasibility and efficacy of HCT in MRD_pos_ patients^11,22,49^. Whether MRD_neg_ patients with HR–VHR profiles could be safely treated without HCT remains to be elucidated in properly designed trials^11^ given the highly complex prognostic interactions that are being disclosed. Other study limitations, common to phase 2 trials in adult ALL, were a non-randomized design that precluded drawing definite conclusions on key aspects of risk-oriented therapy, the reliance on historical controls that cannot match the precision of a randomized comparison of treatment results, and the lack of recognition of novel, highly adverse subsets such as early thymic precursor ALL^52^, Ph-like (*BCR-ABL1*-like) ALL^53^, and others.

Nevertheless, taking these results as a starting point for future research, we wish to remark that this improved strategy was not curative for many patients within the broad age range considered for several reasons, including toxicity. In addition to MRD, a deeper characterization of ALL genetics and biology would allow us to recognize novel HR entities and assign better risk scores, increasing the value of risk-adapted therapy as in the adult UK and other studies^21,23,51,54^. The most rewarding aspect of our study was an MRD_neg_ condition detectable from the end of induction onwards. Such a highly favorable status, achieved in 45% of evaluable study patients, may therefore become a primary therapeutic endpoint. In contrast, the survival rate of VHR MRD_pos_ patients was barely above 30% according to treatment intention and despite the wish to undergo transplantation early on. In the most advanced risk models, all significant prognostic variables can be weighted and integrated through dedicated software into treatment algorithms that predict the probability of failing chemotherapy, HCT, or both, with a goal of establishing priorities among traditional and new experimental therapies. The inclusion of new agents, immunotherapy, and targeted therapy into standard chemotherapy backbones is the next fundamental step to strengthen the whole risk-oriented strategy^2,55^, in association with systematic drug sensitivity screening to reveal unexpected vulnerabilities in scarcely responsive subsets with a poor outcome (e.g., resistance or relapse)^2^. Along these lines, we subsequently incorporated blinatumomab and ponatinib into new protocols for Ph− B-ALL (NCT03367299) and Ph-like ALL^56^.

## Supplementary information

Supplemental files

## References

[1] Bassan R, Hoelzer D (2011). Modern therapy of acute lymphoblastic leukemia. J. Clin. Oncol..

[2] Bassan R, Bourquin JP, DeAngelo DJ, Chiaretti S (2018). New approaches to the management of adult acute lymphoblastic leukemia. J. Clin. Oncol..

[3] Siegel SE (2018). Pediatric-inspired treatment regimens for adolescents and young adults with Philadelphia chromosome-negative acute lymphoblastic leukemia: a review. JAMA Oncol..

[4] Carobolante F, Chiaretti S, Skert C, Bassan R (2020). Practical guidance for the management of acute lymphoblastic leukemia in the adolescent and young adult population. Ther. Adv. Hematol..

[5] Bassan R (2017). Minimal residual disease assessment and risk-based therapy in acute lymphoblastic leukemia. Clin. Lymphoma Myeloma Leuk..

[6] Della Starza I (2019). Minimal residual disease in acute lymphoblastic leukemia:technical and clinical advances. Front. Oncol..

[7] Soverini S, Bassan R, Lion T (2019). Treatment and monitoring of Philadelphia chromosome-positive leukemia patients: recent advances and remaining challenges. J. Hematol. Oncol..

[8] Rafei H, Kantarjian HM, Jabbour EJ (2020). Targeted therapy paves the way for the cure of acute lymphoblastic leukaemia. Br. J. Haematol..

[9] Brüggemann M (2006). Clinical significance of minimal residual disease quantification in adult patients with standard-risk acute lymphoblastic leukemia. Blood.

[10] Gökbuget N (2012). Adult patients with acute lymphoblastic leukemia and molecular failure display a poor prognosis and are candidates for stem cell transplantation and targeted therapies. Blood.

[11] Goekbuget N (2017). Evaluation of minimal residual disease (MRD) and MRD-based treatment decisions in Ph/BCR-ABL-negative adult acute lymphoblastic leukemia (ALL): experience from the German Multicenter Study Group for Adult ALL (GMALL). Blood.

[12] Ribera JM (2014). Treatment of high-risk Philadelphia chromosome-negative acute lymphoblastic leukemia in adolescents and adults according to early cytologic response and minimal residual disease after consolidation assessed by flow cytometry: final results of the PETHEMA ALL-AR-03 trial. J. Clin. Oncol..

[13] Ribera J-M (2016). Comparison of efficacy and safety of two types of *E. coli* asparaginase (native or pegylated) for treatment of adult patients with high-risk (HR), Philadelphia (Ph) chromosome-negative ALL included in the prospective MRD-oriented protocol ALL-HR-11 from the Spanish Pethema Group. Blood.

[14] Ribera JM (2020). A pediatric regimen for adolescents and young adults with Philadelphia chromosome-negative acute lymphoblastic leukemia: results of the ALLRE08 PETHEMA trial. Cancer Med..

[15] Hoelzer D (2016). Acute lymphoblastic leukaemia in adult patients: ESMO Clinical Practice Guidelines for diagnosis, treatment and follow-up. Ann. Oncol..

[16] Short NJ (2019). Recommendations for the assessment and management of measurable residual disease in adults with acute lymphoblastic leukemia: a consensus of North American experts. Am. J. Hematol..

[17] Giebel S (2019). Hematopoietic stem cell transplantation for adults with Philadelphia chromosome-negative acute lymphoblastic leukemia in first remission: a position statement of the European Working Group for Adult Acute Lymphoblastic Leukemia (EWALL) and the Acute Leukemia Working Party of the European Society for Blood and Marrow Transplantation (EBMT). Bone Marrow Transplant.

[18] Bassan R (2009). Improved risk classification for risk-specific therapy based on the molecular study of minimal residual disease (MRD) in adult acute lymphoblastic leukemia (ALL). Blood.

[19] Bassan R (2014). Different molecular levels of post-induction minimal residual disease may predict hematopoietic stem cell transplantation outcome in adult Philadelphia-negative acute lymphoblastic leukemia. Blood Cancer J..

[20] Patel B (2010). Minimal residual disease is a significant predictor of treatment failure in non T-lineage adult acute lymphoblastic leukaemia: final results of the international trial UKALL XII/ECOG2993. Br. J. Haematol..

[21] Beldjord K (2014). Oncogenetics and minimal residual disease are independent outcome predictors in adult patients with acute lymphoblastic leukemia. Blood.

[22] Dhédin N (2015). Role of allogeneic stem cell transplantation in adult patients with Ph-negative acute lymphoblastic leukemia. Blood.

[23] Moorman AV (2019). Clinical efficacy of a novel validated prognostic index for trial design in adult acute lymphoblastic leukaemia. HemaSphere.

[24] Bassan R (2015). Randomized trial of radiation-free central nervous system prophylaxis comparing intrathecal triple therapy with liposomal cytarabine in acute lymphoblastic leukemia. Haematologica.

[25] Masson E (1996). Accumulation of methotrexate polyglutamates in lymphoblasts is a determinant of antileukemic effects in vivo. A rationale for high-dose methotrexate. J. Clin. Invest..

[26] Galpin AJ (1997). Differences in folylpolyglutamate synthetase and dihydrofolate reductase expression in human B-lineage versus T-lineage leukemic lymphoblasts: mechanisms for lineage differences in methotrexate polyglutamylation and cytotoxicity. Mol. Pharmacol..

[27] Mantel N, Byar DP (1974). Evaluation of response-time data involving transient states: an illustration using heart-transplant data. J. Am. Stat. Assoc..

[28] Simon R, Makuch RW (1984). A non-parametric graphical representation of the relationship between survival and the occurrence of an event: application to responder versus non-responder bias. Stat. Med..

[29] Lussana F (2016). Achieving molecular remission before allogeneic stem cell transplantation in adult patients with Philadelphia chromosome-positive acute lymphoblastic leukemia: impact on relapse and long-term outcome. Biol. Blood Marrow Transplant.

[30] Curran E, Stock W (2015). How I treat acute lymphoblastic leukemia in older adolescents and young adults. Blood.

[31] Goekbuget N (2013). Significant improvement of outcome in adolescents and young adults (AYAs) aged 15–35 years with acute lymphoblastic leukemia (ALL) with a pediatric derived adult ALL protocol; results of 1529 AYAs in 2 consecutive trials of the German Multicenter Study Group for Adult ALL (GMALL). Blood.

[32] DeAngelo DJ (2015). Long-term outcome of a pediatric-inspired regimen used for adults aged 18-50 years with newly diagnosed acute lymphoblastic leukemia. Leukemia.

[33] Stock W (2019). A pediatric regimen for older adolescents and young adults with acute lymphoblastic leukemia: results of CALGB 10403. Blood.

[34] Toft N (2018). Results of NOPHO ALL2008 treatment for patients aged 1–45 years with acute lymphoblastic leukemia. Leukemia.

[35] Rowe JM (2010). Prognostic factors in adult acute lymphoblastic leukaemia. Br. J. Haematol..

[36] Chiaretti S (2013). Clinico-biological features of 5202 patients with acute lymphoblastic leukemia enrolled in the Italian AIEOP and GIMEMA protocols and stratified in age cohorts. Haematologica.

[37] Moorman AV (2012). The clinical relevance of chromosomal and genomic abnormalities in B-cell precursor acute lymphoblastic leukaemia. Blood Rev..

[38] Huguet F (2009). Pediatric-inspired therapy in adults with Philadelphia chromosome-negative acute lymphoblastic leukemia: the GRAALL-2003 study. J. Clin. Oncol..

[39] Huguet F (2018). Intensified therapy of acute lymphoblastic leukemia in adults: report of the randomized GRAALL-2005 Clinical Trial. J. Clin. Oncol..

[40] Quist-Paulsen P (2020). T-cell acute lymphoblastic leukemia in patients 1-45 years treated with the pediatric NOPHO ALL2008 protocol. Leukemia.

[41] Bassan R (2018). First results of new GIMEMA trial LAL1913 for adult patients with Philadelphia-negative acute lymphoblastic leukemia (Ph- ALL). HemaSphere.

[42] Bassan R (2000). Role of early anthracycline dose-intensity according to expression of Philadelphia chromosome/BCR-ABL rearrangements in B-precursor adult acute lymphoblastic leukemia. Hematol. J..

[43] Pui C-H (2009). Treatment of childhood acute lymphoblastic leukemia without prophylactic cranial irradiation. N. Engl. J. Med..

[44] Larsen EC (2016). Dexamethasone and high-dose methotrexate improve outcome for children and young adults with high-risk B-acute lymphoblastic leukemia: a report from Children’s Oncology Group Study AALL0232. J. Clin. Oncol..

[45] Winter SS (2018). Improved survival for children and young adults with T-lineage acute lymphoblastic leukemia: results from the Children’s Oncology Group AALL0434 methotrexate randomization. J. Clin. Oncol..

[46] Sakura T (2018). High-dose methotrexate therapy significantly improved survival of adult acute lymphoblastic leukemia: a phase III study by JALSG. Leukemia.

[47] Douer D (2014). Pharmacokinetics-based integration of multiple doses of intravenous pegaspargase in a pediatric regimen for adults with newly diagnosed acute lymphoblastic leukemia. J. Clin. Oncol..

[48] DeAngelo DJ (2015). A multicenter phase II study using a dose intensified pegylated-asparaginase pediatric regimen in adults with untreated acute lymphoblastic leukemia: a DFCI ALL Consortium Trial. Blood.

[49] Berry DA (2017). Association of minimal residual disease with clinical outcome in pediatric and adult acute lymphoblastic leukemia: a meta-analysis. JAMA Oncol..

[50] Gupta V, Richards S, Rowe J, Acute Leukemia Stem Cell Transplantation Trialists’ Collaborative Group (2013). Allogeneic, but not autologous, hematopoietic cell transplantation improves survival only among younger adults with acute lymphoblastic leukemia in first remission: an individual patient data meta-analysis. Blood.

[51] O’Connor D (2018). Genotype-specific minimal residual disease interpretation improves stratification in pediatric acute lymphoblastic leukemia. J. Clin. Oncol..

[52] Bond J (2017). Early response-based therapy stratification improves survival in adult early thymic precursor acute lymphoblastic leukemia: a group for research on adult acute lymphoblastic leukemia study. J. Clin. Oncol..

[53] Chiaretti S. et al. BCR/ABL1-like ALL is associated with MRD persistence and poor outcome. First report from the MRD-oriented front-line GIMEMA LAL1913. *Haematologica*, haematol.2020.247973. 10.3324/haematol.2020.247973 (2020) (Online ahead of print). 10.3324/haematol.2020.247973 PMC8168510 32467145

[54] ALLTogether Protocol_Version 1.0_05AUG2019. A treatment study protocol of the ALLTogether Consortium for children and young adults (1–45 years of age) with newly diagnosed acute lymphoblastic leukemia. EUDRACT number: 2018-001795-2018-001738. https://www.nopho.org/welcome/frame.htm (2018).

[55] Jabbour E, Pui CH, Kantarjian H (2018). Progress and innovations in the management of adult acute lymphoblastic leukemia. JAMA Oncol..

[56] Chiaretti S (2018). Rapid identification of BCR/ABL1-like acute lymphoblastic leukaemia patients using a predictive statistical model based on quantitative real time-polymerase chain reaction: clinical, prognostic and therapeutic implications. Br. J. Haematol..

