# Supplementary material for: Updated risk-oriented strategy for acute lymphoblastic leukemia in adult patients 18–65 years: NILG ALL 10/07
Source: Blood Cancer J. 2020 Nov 13;10(11):119. doi: 10.1038/s41408-020-00383-2 (PMC7666128; doi:10.1038/s41408-020-00383-2)
Supplement: Supplementary file 1 — Supplemental files [file 41408_2020_383_MOESM1_ESM.docx]

**Online supplement to manuscript** “Updated risk-oriented strategy for acute lymphoblastic leukemia in adult patients 18-65 years: NILG ALL 10/07”

| **Supplement (S)** | **File** | **Page** |
| --- | --- | --- |
| S1 | ALL diagnosis and subclassification (NILG ALL 10/07 protocol _v5) | 2 |
| S2 | Treatment protocol: synopsis (NILG ALL 10/07 protocol v_5) | 4 |
| S3 | Amendments 1-5 to NILG ALL 10/07 trial | 9 |
| S4 | Preparative regimen and folinic acid rescue for lineage-targeted MTX infusion | 10 |
| S5 | Comparative analysis of baseline characteristics among patients with Ph- ALL in historical (09/00) and current (10/07) NILG studies | 12 |
| S6 | MRD analysis and results for risk classification after TP2-TP4 MRD | 13 |
| S7 | Outcomes of 42 patients with Ph+ ALL | 14 |
| S8 | Cumulative incidence of relapse (CIR, 5-year rates shown) in CR patients with Ph- ALL in current (10/07) and prior (09/00) NILG studies, relative to (A) all CR patients, (B) clinical standard-risk (SR) and (C) high-risk/very high-risk (HR/VHR) CR patients | 15 |
| S9 | Treatment-associated toxicity during BFM-like (BFM) and lineage-targeted HD-MTX (LT-MTX) consolidation courses | 16 |
| S10 | Correlation between lineage-targeted HD-MTX and MTX through plasma levels | 17 |

##

## S1. ALL diagnosis and subclassification (NILG ALL 10/07 protocol _v5)

The early diagnostic work must be completed in 24-48 hours, in order to allow rapid recognition of eligible cases and registration into study, and must provide the clinician with the following informations:

- Diagnosis of ALL with exclusion of AML/other neoplasms
- Diagnosis of ALL subtype

Diagnostic methodology at presentation is based on jont BM/PB analysis through morphology, cytochemistry and immunophenotype. Cytogenetics and molecular biology studies are of course carried out, but their results are known at a later stage and therefore do not assist in the early diagnostic evaluation. These tests must be applied in an integrated and perfectioned fashion as indicated by major consensus papers of FAB, WHO and EGIL groups:

1. JM Bennett et al. Proposals for the classification of the acute leukaemias. *Br J Haematol 1976; 33: 451.*
2. JM Bennett et al. The morphological classification of acute lymphoblastic leukaemia: concordance among observers and clinical correlations. *Br J Haematol 1981; 47: 553*
3. MC Bene et al. Proposals for the immunological classification of acute leukemias. *Leukemia 1995;9:1783.*
4. Consensual European immunophenotyping panels for leukaemia – 6^th^ PCRDT, april 23, 2005. Obtainable at URL [www.leukemia-net.org](http://www.leukemia-net.org), select “diagnostics” in the upper menu bar, select again “ diagnostics” in the new page, left column: standards/SOP’s of Project 10 will appear automatically.
5. ES Jaffe et al. WHO classification of tumours - Tumours of haematopoietic and lymphoid tissue. IARC Press. Lyon 2001.

Because widely employed and promptly applicable to newly diagnosed patients with ALL, FAB terminology will be retained in the current study for general diagnostic purposes and study registration. The new EGIL/WHO diagnostic terminology will be added to each case following the results of immunophenotype, cytogenetics etc.

Diagnostic material and tests:

- A core marrow biopsy is necessary when marrow aspiration is difficult and/or hypocellular.
- In BM/PB samples, ALL is initially identified according to FAB criteria. However, differing from the L3 subtype that is typcally associated with Burkitt leukemia, the distinction between L1 and L2 is currently of little practical relevance.
- For correct identification of ALL features, both BM cells and PB cells should be evaluated.
- By **morphology**/**cytochemistry**, ALL cells are usually Sudan black B (SBB) and myeloperoxydase (MPO) negative, though SBB+ ALL and/or “granular” ALL is occasionally reported.
- The **immunophenotype panel** includes all necessary reagents to obtain the relevant informations and to discriminate ALL from AML/other disease.

**Table S1.1.** ALL diagnostics by immunophenotype

| **Purpose** | **CD antigens (c=cytoplasmic; Tdt=nuclear)** |
| --- | --- |
| Gating of non-erythroid cells and differential vs. nonhematologic | CD45 (pos) |
| Differential vs. AML | cMPO, CD117 (neg, CD117 rare T-lineage pos) vs. cCD22, cCD79a (pos B-lineage)  cCD3 (pos T-lineage) |
| Establish ALL subset  pro-B (B-I)  “common” (B-II)  pre-B (B-III)  T-lineage pro-T (T-I)  T-lineage pre-T (T-II)  T-lineage cortical (T-III)  T-lineage mature (T-IV) | CD19 and/or cCD79a and/or cCD22plus CD10 (>10%)plus cIgM+ cCD3 and CD7  plus CD2 and/or CD5 and/or CD8  plus CD1a+ plus CD3+, CD1a- |
| Additional markers | TdT CD24 (B-lineage)  anti-TCR (T-lineage)  CD34, CD13, CD33, CD15, anti-MPO, CD64, CDw65 (stem cell/myeloid) |

Cases with <25% lymphoid blast cells in the bone marrow and a definite histopathologic diagnosis of lymphoblastic lymphoma in a lymphnode or other extramedullary tissue are better referred to as such (LL).

Data from morphological and immunophenotypic assessment will be integrated by cytogenetics (and FISH) plus molecular biology tests, in order to indentify specific ALL syndromes within each immunophenotypic subgroup:

**Cytogenetics, FISH and molecular biology**

The following abnormalities will be looked for and registered for the purposes of the current study (standard banding technique or FISH or molecular biology are considered equivalent):

- t(9;22) or *BCR-ABL1* rearrangement
- t(4;11) or *AF4-MLL* rearrangement
- t(1;19) or *PBX/E2A* rearrangement
- t(12;21) or *TEL-AML1* rearrangement
- -7, +8, del6q, t(8;14), other MLL rearrangement at 11q23, low hypodiploid with 30-39 chromosomes, near triploid with 60-78 chromosomes, Complex with >5 unrelated clonal abnormalities
- Hyperdiploid (>50 chromosomes)
- Other

**S2.** Treatment protocol: synopsis (NILG ALL 10/07 protocol _v5).

# SYNOPSIS

| **TITLE** | A randomized pilot study on Central Nervous System (CNS) prophylaxis with standard intrathecal therapy or liposome-encapsulated cytarabine (DepoCyte), together with an improved Minimal Residual Disease (MRD)-oriented induction/consolidation strategy in adult patients with Acute Lymphoblastic Leukemia (ALL). (Protocol NILG-ALL no. 10/07) |
| --- | --- |
| **PROTOCOL VERSION** | No. 5, dated 1 June 2011. |
| **PATIENTS** | Adult patients with ALL as per inclusion/exclusion criteria. All patients aged 18+ years will be included in the ALL Prospective Register. |
| **STUDY DESIGN** | **NILG-ALL 10/07 Trial**  Multicentric prospective pilot randomized phase II trial on CNS prophylaxis with liposomal cytarabine (DepoCyte) vs. standard intrathecal injections. All patients receive induction/consolidation therapy incorporating lineage-targeted high-dose methotrexate plus other drugs (with additional imatinib in Ph/BCR-ABL+ ALL), for the achievement of an early negative MRD status. The MRD study supports a risk/MRD-oriented final consolidation phase.  **Risk Classification**  Newly diagnosed patients are hierarchically clustered into very high, high and standard risk cases (VHR, HR, SR) using international risk criteria modified according to NILG:   - **VHR (any criterium):** B-precursor: WBC count >100x10^9^/L; adverse cytogenetics/molecular biology such as t(9;22)/BCR-ABL, t(4;11)/MLL rearrangement at 11q23, +8, -7, del6q, t(8;14), low hypodiploidy with 30-39 chromosomes, near triploidy with 60-78 chromosomes, complex with >5 unrelated anomalies. T-precursor: WBC count >100x10^9^/L; early/late non-cortical immunophenotype (CD1a-); adverse cytogenetics/molecular biology (as above). - **HR (any criterium, VHR excluded)**: B-precursor: WBC count >30x10^9^/L; pro-B immunophenotype; complete remission after cycle 2. T-precursor: complete remission after cycle 2. - **SR (all criteria, VHR/HR excluded):** B-precursor: WBC count <30x10^9^/L; T-precursor: WBC count <100x10^9^/L; cortical immunophenotype (CD1a+).   **CNS Prophylaxis**  **Stratification before randomisation**   - by immunophenotype, i.e. B-precursor vs. T-precursor - by risk class, i.e. SR vs. non-SR (using only known factors)   **Randomisation:** intrathecal (IT) CNS prophylaxis with standard triple therapy (TIT, 12 total injections) vs. DepoCyte (6-8 total injections by disease subset). Cranial irradiation is omitted in both arms, and all patients receive the same chemotherapy program including CNS-crossing agents. **Induction/Early Consolidation and MRD Study** Randomised patients receive homogeneous induction/early consolidation chemotherapy, with concurrent MRD analysis at four timepoints (weeks 4, 10, 16 and 22 of induction/consolidation), to optimize risk classification and support risk/MRD-oriented therapy:   - **MRD negative (M-NEG):** negative MRD study (<10^-4^ at timepoints #2 and #3, and negative at timepoint #4) - **MRD positive (M-POS):** positive MRD study (>10^-4^ at timepoints #2 or #3, or positive at timepoint #4)   **MRD/Risk-Oriented Final Therapy**   - **VHR** patients are candidate to an early allogeneic SCT (related/unrelated donor/cord blood; ablative/non-ablative conditioning according to current protocols/guidelines) after CR, regardless MRD study results. - **M-POS** as well as **HR** patients with unknown MRD are allocated to allogeneic SCT after MRD timepoint 2 (M-POS >10^-4^) or MRD timepoint 4 (others). When an allogeneic SCT is not possible, patients complete consolidation and receive autologous-type SCT followed by maintenance. - **M-NEG** as well as **SR** patients with unknown MRD are allocated to maintenance therapy.   **Age-limited therapeutic procedures**: Patients aged >55 years are treated with age-adapted therapy, and when indicated will be included in SCT programs whenever possible and according to performance status and comorbidity. |
| **OBJECTIVES** | To evaluate comparatively toxicity and feasibility of intrathecal DepoCyte vs. triple intrathecal therapy, with a preliminary assessment of efficacy (randomized pilot study); to assess the outcome of patients treated with an updated lineage-targeted and MRD/risk-oriented induction/consolidation strategy.  **CNS prophylaxis trial**  **Primary endpoint (feasibility and toxicity)**  Feasibility: comparative analysis of feasibility of IT DepoCyte vs. TIT in conjunction with an early multidrug and multicycle consolidation regimen including lineage-targeted systemic high-dose methotrexate and cytarabine.  Toxicity: comparative analysis of neuromeningeal toxicity during/after IT injections with DepoCyte vs. TIT, according to Common Toxicity Criteria (CTC) clinical scale and an *ad hoc* CNS toxicity evaluation protocol.  **Secondary endpoint (efficacy):** CNS recurrence: comparative analysis of isolated and combined CNS recurrence following TIT vs DepoCyte prophylaxis in all patients and distinct risk subsets.  **Subsidiary outcome measures for efficacy**   1. Complete remission (CR) 2. Early bone marrow MRD negativity at 4-22 weeks/4 timepoints (as surrogate end-point for long term disease-free survival/DFS) 3. Length of remission (DFS) 4. Overall survival (OS) 5. Cumulative incidence of relapse (CIR) 6. Treatment-related mortality (TRM) 7. DFS, OS, CIR and TRM in different age and risk groups 8. Long-term MRD monitoring   **Safety outcome measures**  Toxicity: all adverse events |
| **SAMPLE SIZE** | 150 patients (see Statistical Aspects). |
| **NUMBER OF CENTRES** | 31 centres: 15 of the Northern Italy Leukemia Group (NILG) network and 16 of Gruppo Italiano Malattie Ematologiche dell’Adulto (GIMEMA). |
| **STUDY POPULATION** | **Inclusion criteria**   1. Age 18-65 years. 2. Diagnosis of untreated ALL with B-/T-precursor phenotype, either *de novo* or secondary to chemo-radiotherapy for other cancer. 3. Full cytological, cytochemical, cytogenetic and immunobiological disease characterization by revised FAB, EGIL and WHO criteria. 4. Bone marrow and peripheral blood sampling for MRD study. 5. ECOG performance status 0-2 or reversible ECOG 3 score following intensive care of complications. 6. Signed informed consent.   **Exclusion criteria**   1. Diagnosis of B-ALL (FAB L3 ALL/Burkitt’s leukemia). 2. Down’s syndrome. 3. Pre-existing, uncontrolled pathology such as cardiac disease (congestive/ischemic, acute myocardial infarction within the past 3 months, untreatable arrythmias, NYHA classes III and IV), severe liver disease with serum bilirubin >3 mg/dL and/or ALT >3 x upper normal limit (unless attributable to ALL), kidney function impairment with serum creatinine >2 mg/dL (unless attributable to ALL), and severe neurological or psychiatric disorder that impairs the patient’s ability to understand and sign the informed consent, or to cope with the intended treatment plan. 4. Known HIV positive serology. 5. Other active hematological or non-hematological cancer with life expectancy <1 year. 6. Pregnancy (fertile women will be advised not to become pregnant while on treatment; and male patients to adopt contraceptive methods), unless therapeutic aborption/early discharge is carried out. |
| **TREATMENT AND**  **STUDY DRUGS** | **CNS prophylaxis (Randomisation)**  Standard TIT or DepoCyte are administered during induction/consolidation/maintenance. CR patients continue CNS prophylaxis until relapse in any site or allogeneic/autologous SCT.  **Standard arm**  **TIT** (triple IT therapy) with methotrexate 12.5 mg, cytarabine 50 mg, dexamethasone 4 mg on:   - days 1 and 15 of induction/consolidation cycles 1, 2 and 8; - day 1 of consolidation cycles 4 and 6; - day 1 of maintenance cycles 2, 3, 4 and 5.   (total no. 12)  Patients with CNS involvement at diagnosis:  TIT with methotrexate 15 mg, cytarabine 75 mg, prednisone 40 mg bi-weekly until CNS remission, followed by weekly x2 and monthly x12 (except during consolidation cycles 3, 5 and 7).  **Experimental arm**  Dexamethasone 4 mg, **DepoCyte** 50 mg IT on:   - day 1 of induction/consolidation cycles 1, 2, 4, 6, 8; - day 15 of induction/consolidation cycles 1 and 8 (only T-ALL); - day 1 of maintenance cycle 2 .   (total DepoCyte no. 6 and 8 for B- and T-lineage ALL/LL, respectively)  Patients with CNS involvement at diagnosis:  Dexamethasone 4 mg, DepoCyte 50 mg IT on days 1 and 15 of induction/consolidation cycles 1 and 8, day 1 of consolidation cycles 2, 4 and 6, and day 1 of maintenance cycles 2, 4 and 6 (total 10 doses in all patients).  **Lineage–Targeted Induction/Consolidation Therapy**  Including subset-specific elements for B-precursor ALL (3x targeted-infusion methotrexate 2.5 g/m^2^), T-precursor ALL (3x targeted-infusion methotrexate 5 g/m^2^), age >55 years (methotrexate reduced to 1.5 g/m^2^), Ph/BCR-ABL+ ALL (imatinib, reduced-intensity chemotherapy), radiation therapy (LL). Patients not in CR after cycles 1-2 are off study. For CR evaluation bone marrow is checked on days 28 and/or 56. Consolidation cycles are administered at 21-28 day intervals.  **Induction/early consolidation therapy**   - *Cycle 1*: prednisone 20 mg/m^2^/bd PO (per os) on days -5 to -1, cyclophosphamide 300 mg/m^2^/d IV (intravenous) on days -3 to –1 (pre-induction); idarubicin 12 mg/m^2^/d IV on days 1 and 2, vincristine 1.4 mg/m^2^/d (max. 2 mg) on days 1, 8, 15 and 22, L-asparaginase (E.Coli) 3.000 U/m^2^ IV on days 8, 10, 12, 15, 17 and 19, dexamethasone 5 mg/m^2^/bd IV on days 1-5, 15-19, G-CSF from day 5 (induction). - *Cycle 2:* idarubicin 12 mg/m^2^/d IV on day 1, cyclophosphamide 1000 mg/m^2^ IV on day 1, dexamethasone 5 mg/m^2^/bd IV/PO on days 1-5, cytarabine 75 mg/m^2^/d IV/SC (subcutaneous) on days 2-5, 6-mercaptopurine 60 mg/m^2^/d PO on days 1-10, G-CSF from day 8 to resolution of absolute neutropenia <1 x10^9^/L. - *Cycles 3,7*: methotrexate 2.5/5 (B/T phenotype) g/m^2^/d IV on day 1 (24-h infusion, folinic acid rescue), cytarabine 2 g/m^2^/bd IV on days 3 and 4, G-CSF from day 8 (collection/cryopreservation of autologous blood stem cells at cycle 3). - *Cycles 4,6:* idarubicin 12 mg/m^2^/d IV on day 1, cyclophosphamide 1000 mg/m^2^ IV on day 1, vincristine 1.4 mg/m^2^/d (max. 2 mg) IV on days 1 and 8, dexamethasone 5 mg/m^2^/bd IV/PO on days 1-5, cytarabine 75 mg/m^2^/d IV/SC (subcutaneous) on days 2-5, 6-mercaptopurine 60 mg/m^2^/d PO on days 1-10, G-CSF from day 8 to resolution of absolute neutropenia <1 x10^9^/L. - *Cycle 5:* methotrexate 2.5/5 (B/T phenotype) g/m^2^/d IV on day 1 (24-h infusion, folinic acid rescue), L-asparaginase (E. Coli) 10.000 U/m^2^ IV on days 3 and 8. - *Cycle 8:* idarubicin 10 mg/m^2^/d IV on days 1 and 8, vincristine 1.4 mg/m^2^/d (max. 2 mg) IV on days 1 and 8, cyclophosphamide 300 mg/m^2^/d IV on days 1-3, dexamethasone 5 mg/m^2^/bd IV/PO on days 1-5, prednisone 20 mg/m^2^/bd PO on days 8-12, G-CSF from neutropenia <0.5 microl to its resolution.   Variations for Ph/BCR-ABL+ ALL:   - *Cycle 1*: imatinib 400 mg/bd PO on days 1-28, idarubicin on day 1 only, L-asparaginase omitted. - *Cycle 2*: imatinib 400 mg/bd PO on days 1-21, idarubicin 10 mg/m^2^, cyclophosphamide 650 mg/m^2^, cytarabine on days 2-5 only, 6-mercaptopurine on days 1-7 only. - *Cycles 3, 7*: imatinib 400 mg/bd PO on days 8-21, methotrexate 1.5 g/m^2^. - *Cycles 4, 6*: imatinib 400 mg/bd PO on days 8-21, idarubicin 10 mg/m^2^, cyclophosphamide 650 mg/m^2^, vincristine on day 1 only, cytarabine on days 2-5 only, 6-mercaptopurine on days 1-7 only. - Cycle 5: imatinib 400 mg/bd PO on days 8-21, methotrexate 1.5 g/m^2^. - *Cycle 8*: imatinib 400 mg/bd PO on days 8-21, idarubicin on day 1 only, cyclophosphamide omitted.   Variations for age >55 years (all):   - *Cycles 3, 7*: methotrexate 1.5 g/m^2^. - *Cycle 5*: methotrexate 1.5 g/m^2^.   **MRD/Risk-Oriented Therapy**  **M-NEG/SR patients: Maintenance (24 4-week cycles)**   - *Cycles 1, 3, 5, 7, 9, 11*: cyclophosphamide 100 mg/m^2^/d PO on days 1-4, 6-mercaptopurine 75 mg/m^2^/d PO on days 8-28, methotrexate 15 mg/m^2^/d PO/IM (intramuscular) on days 8, 15 and 22. - *Cycles 2, 4, 6, 8, 10, 12:* vincristine 1 mg/m^2^ IV on day 1, prednisone 40 mg/m^2^/d PO on days 1-5, 6-mercaptopurine 75 mg/m^2^/d PO on days 8-28, methotrexate 15 mg/m^2^/d PO/IM on days 8, 15 and 22. - *Cycles 13-24:* 6-mercaptopurine 75 mg/m^2^/d PO on days 1-28, methotrexate 15 mg/m^2^/d PO/IM on days 1, 8, 15 and 22.   **M-POS/HR and VHR patients: 1^st^ option Allogeneic SCT**   - *Allogeneic SCT:* first choice option, from sibling/unrelated donor or cord blood. SCT procedure by local guidelines/protocols. SCT timing is by risk class (VHR: early) and MRD study results (positive timepoint 2: early; others: at end of consolidation, with interim maintenance).   **M-POS/HR and VHR patients: 2^nd^ option Autologous SCT with Maintenance (12 4-week cycles)**   - *Autologous SCT*: second choice option if allogeneic SCT not possible (NB: maintenance only if autologous SCT not feasible), with melphalan 100 mg/m^2^/d IV on days 1 and 2, plus unpurged autologous CD34+ blood cells (2-6x10^6^/kg) on day 4, and G-CSF. - *Maintenance cycles 1, 3, 5, 7, 9, 11:* cytarabine 300 mg/m^2^ IV on day 1*,* cyclophosphamide 100 mg/m^2^/d PO on days 1-4, 6-mercaptopurine 75 mg/m^2^/d PO on days 8-28, methotrexate 15 mg/m^2^/d PO/IM on days 8, 15 and 22. - *Maintenance cycles 2, 4, 6, 8, 10, 12:* vincristine 1 mg/m^2^ IV on day 1, prednisone 40 mg/m^2^/d PO on days 1-5, 6-mercaptopurine 75 mg/m^2^/d PO on days 8-28, methotrexate 15 mg/m^2^/d PO/IM on days 8, 15 and 22, idarubicin 10 mg/m^2^ IV on day 1 (cycles 4, 8 and 12 only).   **M-POS/HR and VHR patients excluded from SCT: 3^rd^ option Maintenance (24 4-week cycles)**   - *Maintenance cycles 1, 3, 5, 7, 9, 11:* cytarabine 300 mg/m^2^ IV on day 1*,* cyclophosphamide 100 mg/m^2^/d PO on days 1-4, 6-mercaptopurine 75 mg/m^2^/d PO on days 8-28, methotrexate 15 mg/m^2^/d PO/IM on days 8, 15 and 22. - *Maintenance cycles 2, 4, 6, 8, 10, 12:* vincristine 1 mg/m^2^ IV on day 1, prednisone 40 mg/m^2^/d .PO on days 1-5, 6-mercaptopurine 75 mg/m^2^/d PO on days 8-28, methotrexate 15 mg/m^2^/d PO/IM on days 8, 15 and 22, idarubicin 10 mg/m^2^ IV on day 1 (cycles 4, 8 and 12 only). - *Cycles 13-24:* 6-mercaptopurine 75 mg/m^2^/d PO on days 1-28, methotrexate 15 mg/m^2^/d PO/IM on days 1, 8, 15 and 22.   Variations for Ph/BCR-ABL+ ALL:   - *Maintenance cycles 1, 3, 5, 7, 9, 11:* imatinib 400 mgb/d PO on days 1-28, 6-mercaptopurine and methotrexate on days 1-14, cytarabine and cyclophosphamide omitted. - *Maintenance cycles 2, 4, 6, 8, 10, 12*: imatinib 400 mg/bd PO on days 1-28, 6-mercaptopurine on days 8-14, methotrexate on day 8. - *Maintenance cycles 13*-*24+* : imatinib 400 mg/bd PO on days 1-28, 6-mercaptopurine 75 mg/m^2^/d PO on days 1-14, methotrexate 15 mg/m^2^/d PO/IM on days 1 and 8. After cycle 24: imatinib 400 mg/bd PO until relapse. |
| **STATISTICAL**  **ASPECTS** | The primary end-point for the calculation of sample size is the demonstration of safety and tolerability of DepoCyte in the prevention of CNS relapse, and the achievement of an early MRD negative status (i.e. at 10-22 weeks) in CR.  **Current clinical expectations**  Prior NILG studies indicate: A) CR rate of 85%, B) risk of isolated CNS recurrence of 1% for B-lineage ALL (75% of the cases) and 7,5% for T-lineage ALL (25% of the cases), C) early relapse rate of 10% in SR group, 50% in HR/VHR subsets, and D) early MRD negativity rate of 42% associated with a DFS of 75%.  **SAMPLE SIZE**  **CNS prophylaxis trial**  A reasonable estimate of the number of patients who can be recruited in this study by NILG is approximately 50 per year. The expected global failure proportion of current CNS prophylaxis is approximately 2,5%. Based on preliminary results of Interim Analysis (see Statistical Aspects), a recruitment period of additional 2 years will allow to increase study sample size and randomize 150 patients in the CNS pilot trial, appearing to warrant a comparative outcome analysis with historical data as regards the new risk/MRD-based treatmet study. Due to the rarity of the disease as well as of the neurological complications, a formal sample size calculation according to "classical" statistical tenets would give rise to a disproportionately high number of patients to be recruited. Therefore it has been decided to privilege the collection of standardized, methodologically sound information in the context of a formal randomized clinical trial over data gathered through "pure" empirism. In this design, the comparative description of drug-associated neurotoxicity will be a major study endpoint.  **Remission induction and consolidation/MRD study**  85% of patients are expected to enter CR For the postremission consolidation/MRD study a sample size of 150 patients achieves a power > 90% to detect a difference (P0 - P1) of -0.1800 between the null hypothesis that the population proportion is 42% and the alternative hypothesis that the population proportion is 60% using a two-sided, binomial hypothesis test with a target significance level of 0.05 (the actual significance level is 0.04615) |
| **LENGTH OF STUDY** | Starting January 2008, 4 years (or more if necessary) for patient enrollment (150 patients) and a minimum of 1 year of follow-up from the date of randomization of the last patient. Accordingly, study results should be amenable to final analysis in 2013. Patient enrolled in the outcome studies are followed-up annually for survival and disease status. |

**S3.** Amendments 1-5 to NILG ALL 10/07 trial.

| **Protocol version** | **Dated** | **Study amendments** | |
| --- | --- | --- | --- |
|  |  | **Issue(s)** | **Action(s)** |
| 1 | November 15, 2007 | - | - |
| 2 | February 18, 2008 | - IT CNS prophylaxis trial: risk of cumulative CNS toxicity due to close exposure (≤ 14 days) to systemic HD MTX and cytarabine at C3,5 and 7 | - No IT liposome-encapsulated cytarabine on day 15 of C2,4,6 in CNS+ patients |
|  |  | - Supportive care | - Start of G-CSF with ANC <0.5 x10^9^/L at C2,4,6 and 8 - Improved instructions for FA rescue following HD MTX at C3,5 and 7 |
| 3 | January 26, 2009 | - Prolonged myelotoxicity with infectious complications (C2,4,6; Ph- ALL) | - Dosing of 6-mercaptopurine and cytarabine guided by ANC on day 8 of each course |
| 4 | June 12, 2009 | - Prolonged myelotoxicity with deaths by infections after C2,4,6 - Antifugal prophylaxis during CR induction (C1) | - Dosing of 6-mercaptopurine and cytarabine reduced from 14 to 10 days, and from 8 to 4 days, respectively - IV liposomal amphotericin B 50 mg on alterate days suggested |
|  |  | - IT CNS prophylaxis trial: risk of neurotoxicity and sample size of randomized trial | - Addition of IT dexamethasone 4 mg to IT liposome-encapsulated cytarabine - Total number trial patients increased from 100 to 150 |
| 5 | June 1, 2011 | - Sample size of study patients after closure of IT CNS prophylaxis trial | - Total number of study patients increased from 150 to 200 |

IT, intrathecal; C, course; CNS central nervous system; HD, high dose; G-CSF, granulocyte colony-stimulating factor; ANC, absolute neutrophil count; FA, folinic acid; MTX, methotrexate; CR, complete remission; IV, intravenous

**S4.** Preparative regimen and folinic acid rescue for lineage-targeted MTX infusion.

***Targeted Infusion Methotrexate Protocol***

***General***

- *No active infection, normal kidney and liver function, no pleural/abdominal effusions*

***DAY –2***

- *Ensure adequate fluid intake (3 l/d) with allopurinol 300 mg/d PO*

***DAY –1***

- *Start* ***IV hydration*** *with 5% dextrane solution 3000 ml/m^2^/d + KCl 35 mEq/m2/d + NaHCO3 60 mEq/m^2^/d*
- *Maintain* ***urinary output*** *>75 ml/h by adding furosemide 20 mg IV/bd (or more if weight gain >1 kg).*

***MTX Infusion and Folinic Acid (FA) Rescue***

***DAY 1***

- *Continue* ***IV hydration****, keeping* ***urinary pH >7*** *with more NaHCO3 (1 mEq/kg), until 48 h from end of MTX (h 72)*
- ***Urinary output*** *>75 ml/h by adding furosemide 20 mg IV/bd (or more if weight gain >1 kg).*

**FA rescue starts 18 h from end of MTX infusion (or at end if MTX level is >150 microM/l) and continues until MTX is <0.25 microM/l. Each FA dose is based on MTX level measured 6 h earlier.**

***h 0 (18.00)……****START* ***MTX*** *(1/10 total dose over 0.5 h, 9/10 over 23.5 h)*

***h 8 (2.00)……. MTX level***

***h 24 (18.00)…..****END* ***MTX***  ***MTX level,*** *creatinine*

***>150 microM/l* FA 37,5 mg/m^2^ IV***

***FA 37,5 mg/m^2^ IV DAY +1 FA 37,5 mg/m^2^ IV***

***h 36 (6.00) ……………..MTX level*………………***

***h 42 (12.00)…………..MTX level****…………* ***FA*** ***mandatory (1^st^) 🗹***

***as above. 20 mg/m^2^/IV (MTX <2)***

***30 mg/m^2^/IV (MTX 2-2.99)***

***37.5 mg/m^2^ IV (MTX 3-5) (MTX x body weight/2) mg IV (MTX >5)***

***h 48 (18.00)……………MTX level…………….. FA mandatory (2^nd^) 🗹***

***as above*** ***as above***

***DAY +2***

***h 54 (24.00)…………….MTX level………………FA mandatory (3^rd^) 🗹***

***h 54 (24.00)…………….MTX level………………FA mandatory (3^rd^) 🗹***

***as above*** ***as above***

***h 60 (6.00)………………MTX level………………FA***

***>0.25 microM/l as*** ***above***

*(****as above****)*

***h 66 (12.00)……****Continue above schedule……...****FA***

*every 6 hours until* ***MTX level*** ***<0.25 microM/l***

***Warnings***

- ***NB 1*** *MTX level at h 42 and/or later >5 microM/l:*

***IV hydration*** *4500 ml/m^2^/24 h, urinary pH >7 (NaHCO3 1 mEq/kg), repeatable*

- ***NB 2*** *MTX intoxication with systemic/CNS symptoms, urinary pH <6, kidney failure:*

***Carboxypeptidase-G2 (Voraxaze) 50 U/kg IV*** *obtainable from ADIENNE Pharma & Biotech (http://www.adienne.com/it/, 24128 Bergamo, Via Broseta 64/B; tel. 035264206, e-mail* [adienne@adienne.com](mailto:adienne@adienne.com)*.*

**S5.** Comparative analysis of baseline characteristics among patients with Ph- ALL in historical (09/00) and current (10/07) NILG studies.

|  | **Study 09/00**  **(n=304)** | **Study 10/07**  **(n=161)** | **P value** |
| --- | --- | --- | --- |
|  |  |  |  |
| **Age** (years), median (range) | 35 (15-67) | 40 (17-67) | 0.08 |
| ≤55, n (%) | 256 (84.2) | 135 (83.9) | 0.92 |
| >55, n (%) | 48 (15.8) | 26 (16.1) |  |
| **Gender** (male), n (%) | 173 (56.9) | 94 (58.4) | 0.76 |
| **Hemoglobin** (g/dl), median (range) | 9.5 (2.4-16) | 9.8 (3.4-16.8) | 0.33 |
| **WBC** (10^9^/l), median (range) | 15.2 (0.4-900) | 8.6 (0.4-1021) | 0.01 |
| >100, n (%) | 53 (17.4) | 25 (15.5) | 0.60 |
| **BM blasts** (%), median (range) | 90 (30-100) | 90 (12-100) | 0.001 |
| **PB blasts** (%), median (range) | 55 (0-100) | 47 (0-100) | 0.11 |
| **Platelets** (10^9^/l), median (range) | 45 (3.6-420) | 60.5 (5-450) | 0.03 |
| **Hepatomegaly**, n (%) | 103 (34) | 30 (18.6) | 0.0005 |
| **Splenomegaly**, n (%) | 134 (44.5) | 46 (28.6) | 0.0009 |
| **Lymphadenopathy**, n (%) | 104 (34.6) | 32 (19.9) | 0.001 |
| **Mediastina mass**, n (%) | - | 19 (11.8) | - |
| **CNS involvement**, n (%) | 17 (5.8) | 3 (1.9) | 0.05 |
| **Immunophenotype,** n (%) |  |  |  |
| pro-B | 57 (19.6) | 27 (16.9) | 0.48 |
| common-B | 115 (39.5) | 62 (38.8) | 0.87 |
| pre-B | 37 (12.7) | 27 (16.9) | 0.23 |
| pro-T | 1 (0.3) | 6 (3.8) | 0.009 |
| pre-T | 23 (7.9) | 13 (8.1) | 0.93 |
| cortical-T | 47 (16.2) | 21 (13.1) | 0.39 |
| mature-T | 11 (3.8) | 4 (2.5) | 0.47 |
| **Cytogenetics/genetics**, n(%) |  |  |  |
| normal | 107 (35.7) | 77 (47.8) | 0.01 |
| adverse | 54 (18) | 36 (22.4) | 0.26 |
| non-adverse | 62 (20.7) | 25 (15.5) | 0.18 |
| not known | 77 (25.7) | 23 (14.3) | 0.005 |
| **Risk stratification**, n (%) |  |  | 0.04 |
| standard-risk | 170 (55.9) | 73 (45.3) |  |
| high-risk | 20 (6.6) | 20 (12.4) |  |
| very high-risk | 112 (36.8) | 68 (42.2) |  |

**S6.** MRD analysis for risk classification of study patients, based on TP2-TP4 MRD as per study design. Data shown according to ALL subset and clinical risk stratification (SR, standard risk; HR, high risk; VHR, very high risk). Final risk classification was used for allocation of SR and HR patients to either maintenance chemotherapy or allogeneic HCT. VHR patients were allocated to HCT independently of MRD study results. The number of patients evaluable at TP3 (week 16) and TP4 (week 22) decreased owing to some early study losses and the early shift to allogeneic HCT of patients with VHR characteristics and/or TP2 MRD ≥10^-4^.

|  | **All patients**  **(n=140)** | **T-ALL** | | | **B-ALL** | | | |
| --- | --- | --- | --- | --- | --- | --- | --- | --- |
|  |  | All (n=43) | SR (n=11) | VHR (n=32) | All (n=97) | SR (n=52) | HR (n=16) | VHR (n=29) |
| **TP2 MRD**, n (%)  evaluable  negative  <10^-4^  ≥10^-4^ | 106  64 (60.4)  11 (10.4)  31 (29.2) | 36 (83.7)  22 (61.1)  5 (13.9)  9 (25.0) | 10 (90.9)  8 (80.0)  1 (10.0)  1 (10.0) | 26 (81.3)  14 (53.8)  4 (15.4)  8 (30.8) | 70 (72.2)  42 (60.0)  6 (8.6)  22 (31.4) | 37 (71.2)  25 (67.6)  4 (10.8)  8 (21.6) | 14 (87.5)  6 (42.9)  2 (14.3)  6 (42.9) | 19 (65.5)  11 (57.9)  0 (0.0)  8 (42.1) |
| **TP3 MRD**, n (%)  evaluable  negative  <10^-4^  ≥10^-4^ | 55  38 (69.1)  7 (12.7)  10 (18.2) | 16 (37.2)  12 (75.0)  1 (6.3)  3 (18.8) | 8 (72.7)  6 (75.0)  1 (12.5)  1 (12.5) | 8 (25.0)  6 (75.0)  0 (0.0)  2 (25.0) | 39 (40.2)  26 (66.7)  6 (15.4)  7 (17.9) | 29 (55.8)  19 (65.5)  5 (17.2)  5 (17.2) | 5 (31.3)  5 (100.0)  0 (0.0)  0 (0.0) | 5 (17.2)  2 (40.0)  1 (20.0)  2 (40.0) |
| **TP4 MRD**, n (%)  evaluable  negative  <10^-4^  ≥10^-4^ | 58  41 (70.7)  8 (13.8)  9 (15.5) | 14 (32.6)  10 (71.4)  1 (7.1)  3 (21.4) | 8 (72.7)  6 (75.0)  1 (12.5)  1 (12.5) | 6 (18.8)  4 (66.7)  0 (0.0)  2 (33.3) | 44 (45.4)  31 (70.5)  7 (15.9)  6 (13.6) | 30 (57.7)  23 (76.7)  4 (13.3)  3 (10.0) | 7 (43.8)  5 (71.4)  1 (14.3)  1 (14.3) | 7 (24.1)  3 (42.9)  2 (28.6)  2 (28.6) |
| **MRD risk model^1^**, n (%)  Evaluable  MRD_pos_  MRD_neg_ | 109 (77.9)  41 (37.6)  68 (62.4) | 36 (83.7)  10 (27.8)  26 (72.2) | 10 (90.9)  2 (20.0)  8 (80.0) | 26 (81.3)  8 (30.8)  18 (69.2) | 73 (75.3)  31 (42.5)  42 (57.5) | 39 (75.0)  12 (30.8)  27 (69.2) | 14 (87.5)  8 (57.1)  6 (42.9) | 20 (69.0)  11 (55.0)  9 (45.0) |

Abbreviations: CR, complete remission; NR, no response/refractory; ED, early death; TP, time-point; MRD, minimal residual disease (neg, negative; pos, positive; u/k, unknown); HCT, hematopoietic cell transplantation

^1^as applied to all MRD evaluable patients with SR, HR and VHR Ph- ALL

**S7.**  Outcomes of 42 adult patients with Ph- ALL. Shown is overall survival of all 42 study patients, estimated at 45% and 39% at 5 and 10 years, respectively (A); disease-free survival, estimated at 37% and 34% (B); cumulative incidence of relapse, estimated at 39% at 5+years(C); and treatment-related mortality (TRM), estimated at 26% and 29% (D).


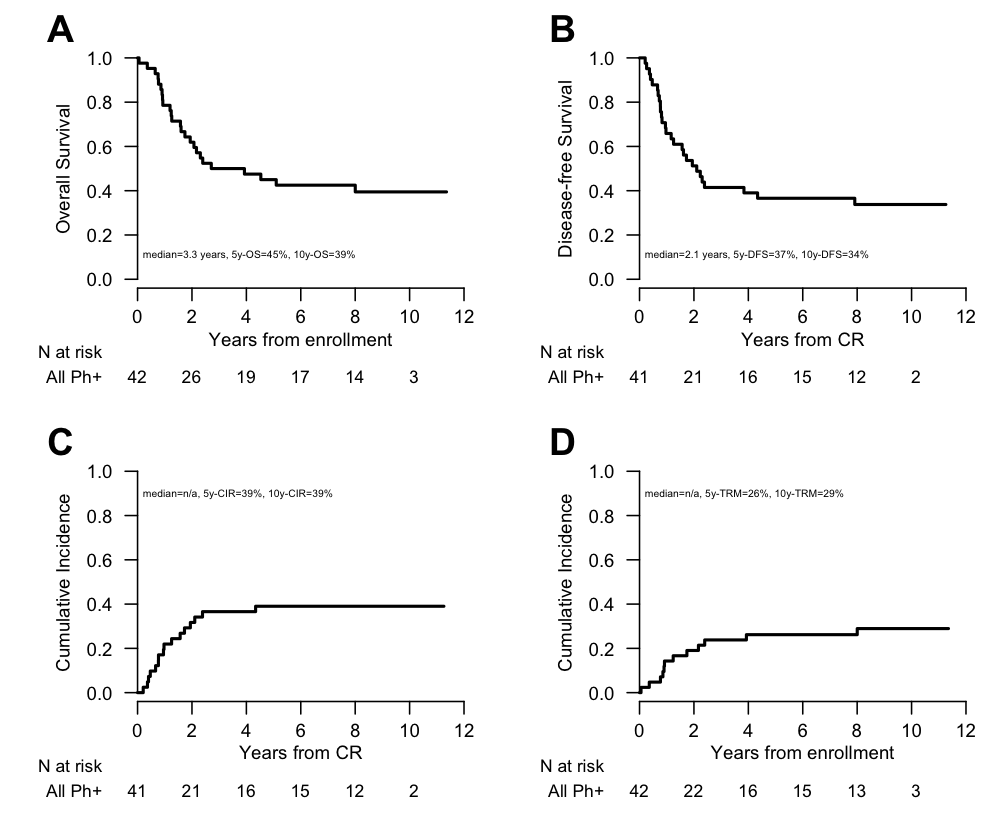


**S8**. Cumulative incidence of relapse (CIR, 5-year rates shown) in CR patients with Ph- ALL in current (10/07) and prior (09/00) NILG studies, relative to (A) all CR patients, (B) clinical standard-risk (SR) and (C) high-risk/very high-risk (HR/VHR) CR patients.

**
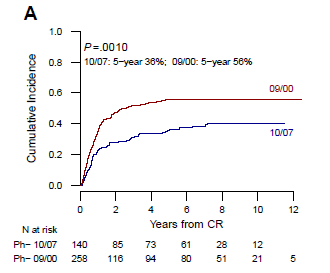
**

**
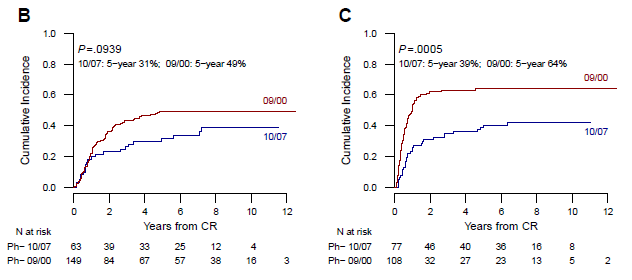
**

**S9.** Treatment-associated toxicity registered during BFM-like (BFM) and lineage-targeted HD-MTX (LT-MTX) consolidation courses. Data are shown according to patient age ≤ vs. >55 years. Only severe adverse events with CTC grading >2 are reported.

|  | **All**^1^ | **BFM-like** | | **LT-MTX^2^** | |
| --- | --- | --- | --- | --- | --- |
|  |  | **Age ≤55** | **Age >55** | **Age ≤55** | **Age >55** |
| No. of patients treated | 137 | 122 | 15 | 119 | 14 |
| No. of courses | 634 | 301 | 34 | 269 | 30 |
| **Hematologic toxicity (days to recover), median (range)** | | | | | |
| Neutrophil count >0.5 (x10^9^/L) | 15 (0-45) | 17 (0-33) | 17 (0-45) | 14 (0-30) | 14 (0-18) |
| Platelet count >20 (x10^9^/L) | 15 (0-170) | 15 (0-170) | 0 (0-31) | 15 (0-72) | 16 (0-22) |
| **Fever and infections (clinical picture and etiology), no. (%)** | | | | | |
| Fever > 38 °C | 103 (16.2) | 51 (16.9) | 10 (29.4) | 33 (12.3) | 9 (30.0) |
| Fever of unknown origin | 48 (7.6) | 28 (9.3) | 4 (11.8) | 13 (4.8) | 3 (10.0) |
| Bacteremia | 28 (4.4) | 10 (3.3) | 2 (5.9) | 12 (4.5) | 4 (13.3) |
| Sepsis | 15 (2.4) | 8 (2.6) | 1 (2.9) | 6 (2.2) | 0 |
| Pneumonia | 15 (2.4) | 9 (3.0) | 4 (11.8) | 1 (0.4) | 1 (3.3) |
| Other involved site | 42 (6.6) | 15 (5.0) | 1 (2.9) | 21 (7.8) | 5 (16.7) |
| *Gastrointestinal system* | 14 (2.2) | 3 (1.0) | 1 (2.9) | 8 (3.0) | 2 (6.7) |
| *Skin* | 29 (4.6) | 12 (4.0) | 0 | 14 (5.2) | 3 (10.0) |
| *Urinary system* | 1 (0.1) | 0 | 0 | 1 (0.4) | 0 |
| Bacterial | 49 (7.7) | 19 (6.3) | 3 (8.8) | 20 (7.4) | 7 (23.3) |
| Gram+ | 29 (4.6) | 12 (4.0) | 2 (5.9) | 10 (3.7) | 5 (16.7) |
| Gram- | 21 (3.3) | 7 (2.3) | 1 (2.9) | 11 (4.1) | 2 (6.7) |
| Fungal, candida spp. | 2 (0.3) | 0 | 1 (2.9) | 1 (0.4) | 0 |
| Parasitic | 1 (0.1) | 1 (0.3) | 0 | 0 | 0 |
| Viral | 14 (2.2) | 5 (1.7) | 0 | 9 (3.3) | 0 |
| Unknown | 69 (10.9) | 38 (12.6) | 7 (20.6) | 20 (7.4) | 4 (13.3) |
| **Grade III-IV non-hematological toxicity (type/organ), no. (%)** | | | | | |
| Hemorrhage | 7 (1.1) | 4 (1.3) | 1 (2.9) | 2 (0.7) | 0 |
| Cardiovascular system | 7 (1.1) | 2 (0.7) | 1 (2.9) | 4 (1.5) | 0 |
| Coagulation | 6 (0.9) | 2 (0.7) | 0 | 4 (1.5) | 0 |
| Hepatobiliary system | 29 (4.6) | 14 (4.6) | 2 (5.9) | 13 (4.8) | 0 |
| Metabolism | 5 (0.8) | 2 (0.7) | 0 | 2 (0.7) | 1 (3.3) |
| Kidney | 3 (0.5) | 1 (0.3) | 1 (2.9) | 0 | 1 (3.3) |
| Amylase | 1 (0.1) | 1 (0.3) | 0 | 0 | 0 |
| Central/peripheral nervous system | 43 (6.8) | 38 (12.6) | 4 (11.8) | 0 | 1 (3.3) |
| Gastrointestinal system | 11 (1.7) | 1 (0.3) | 1 (2.9) | 7 (2.6) | 2 (6.7) |
| Lungs | 1 (0.1) | 1 (0.3) | 0 | 0 | 0 |
| Allergy | 2 (0.3) | 0 | 0 | 2 (0.7) | 0 |
| **Treatment-related deaths, no. (%)^3^** | 3 (0.5) | 1 (0.3) | 2 (5.9) | 0 | 0 |

^1^cumulative analysis from all CR patients and all consolidation courses (at least 1 administered)

^2^fixed-dose MTX (1.5 g/m^2^) in patients >55 years

^3^vs. total no. of patients treated

**S10.** Correlation between lineage-targeted HD-MTX and MTX through plasma levels. MTX dosing: B-ALL 2.5 g/m^2^; T-ALL 5 g/m^2^; patients aged >55 years 1.5 g/m^2^. MTX through plasma levels were assessed at 8 h and 24 h since start of MTX infusion. Targeted drug levels were 33 and 65 micromol/l in B- and T-ALL, respectively.

|  | **All** | **B-ALL** | | **T-ALL** | |
| --- | --- | --- | --- | --- | --- |
|  |  | **Age ≤55** | **Age >55** | **Age ≤55** | **Age >55** |
| No. of patients treated | 168 | 106 | 20 | 40 | 2 |
| No. of courses | 352 | 226 | 41 | 80 | 5 |
| **8-h MTX-emia** (micromol/l), |  |  |  |  |  |
| median (IQR range) | 34 (22-58) | 33 (23-45) | 24 (13-30) | 69 (28-97) | 29 (20-31) |
| mean (±SD) | 45 (±45) | 41 (±45) | 22 (±15) | 68 (±50) | 23 (±15) |
| **24-h MTX-emia** (micromol/l), |  |  |  |  |  |
| median (IQR range) | 34 (15-55) | 32 (12-43) | 25 (9-34) | 66 (32-86) | 30 (28-39) |
| mean (±SD) | 39 (±34) | 32 (±25) | 28 (±24) | 66 (±47) | 33 (±10) |

IQR, interquartile; SD, standard deviation
